# A systematic review of clinical practice guidelines and recommendations for the management of pain, sedation, delirium and iatrogenic withdrawal syndrome in pediatric intensive care

**DOI:** 10.3389/fped.2023.1264717

**Published:** 2023-10-06

**Authors:** Ibo MacDonald, Silvia Alvarado, Mark T. Marston, Luz Gomez Tovar, Vivianne Chanez, Eva Favre, Ying Gu, Alexia Trombert, Maria-Helena Perez, Anne-Sylvie Ramelet

**Affiliations:** ^1^ Institute of Higher Education and Research in Healthcare, Faculty of Biology and Medicine, University of Lausanne, Lausanne, Switzerland; ^2^ Pediatric Intensive Care Unit, Department Woman-Mother-Child, Lausanne University Hospital, Lausanne, Switzerland; ^3^ Faculty of Health, Universidad Surcolombiana, Neiva, Colombia; ^4^ Department Adult Intensive Care, Lausanne University Hospital, Lausanne, Switzerland; ^5^ Nursing Department, Children's Hospital of Fudan University, Shanghai, China; ^6^ Medical Library, Lausanne University Hospital and University of Lausanne, Lausanne, Switzerland

**Keywords:** delirium, practice guideline, iatrogenic withdrawal syndrome, pain, sedation, intensive care units, pediatric, critical care

## Abstract

**Introduction:**

This systematic review aimed to evaluate the quality of clinical practice guidelines (CPGs) and recommendations for managing pain, sedation, delirium, and iatrogenic withdrawal syndrome in pediatric intensive care (PICU). The objectives included evaluating the quality of recommendations, synthesizing recommendations, harmonizing the strength of the recommendation (SoR) and the certainty of evidence (CoE), and assessing the relevance of supporting evidence.

**Methods:**

A comprehensive search in four electronic databases (Medline, Embase.com, CINAHL and JBI EBP Database), 9 guideline repositories, and 13 professional societies was conducted to identify CPGs published from January 2010 to the end of May 2023 in any language. The quality of CPGs and recommendations was assessed using the AGREE II and AGREE-REX instruments. Thematic analysis was used to synthesize recommendations, and the GRADE SoR and CoE harmonization method was used to interpret the credibility of summary recommendations.

**Results:**

A total of 18 CPGs and 170 recommendations were identified. Most CPGs were of medium-quality, and three were classified as high. A total of 30 summary recommendations were synthesized across each condition, focused on common management approaches. There was inconsistency in the SoRs and CoE for summary recommendations, those for assessment showed the highest consistency, the remaining were conditional, inconsistent, inconclusive, and lacked support from evidence.

**Conclusion:**

This systematic review provides an overview of the quality of CPGs for these four conditions in the PICU. While three CPGs achieved high-quality ratings, the overall findings reveal gaps in the evidence base of recommendations, patient and family involvement, and resources for implementation. The findings highlight the need for more rigorous and evidence-based approaches in the development and reporting of CPGs to enhance their trustworthiness. Further research is necessary to enhance the quality of recommendations for this setting. The results of this review can provide a valuable foundation for future CPG development.

**Systematic Review Registration:**

https://www.crd.york.ac.uk/prospero/display_record.php?RecordID=274364, PROSPERO (CRD42021274364).

## 1. Introduction

Management of pain and sedation in pediatric intensive care patients remains suboptimal, with under-reported and under-treated pain (1–3). Prevalence rates of pain can reach up to 47% (2), with instances of under-sedation (10.6%) and over-sedation (31.8%) (4). Inappropriate pain and sedation management have negative physiological and psychological consequences (5). Prolonged administration of analgesics and sedatives increases the risk of delirium and iatrogenic withdrawal syndrome (IWS) (6–8), emphasizing the need for appropriate assessment and treatment for these conditions. To accomplish this, healthcare professionals (HCPs) should be able to rely on evidence-based best practice recommendations.

Despite available recommendations for pain, sedation, delirium and IWS management, their implementation internationally and across European pediatric intensive care units (PICUs) is inconsistent and highly heterogenous (9–11). Bridging this gap can be achieved through systematic adoption of evidence-based recommendations found in clinical practice guidelines (CPGs). Clinical practice guidelines are “statements that include recommendations intended to optimize patient care that are informed by a systematic review of evidence and an assessment of benefits and harms of alternative care options” (p. 6) (12). They serve as a guidance document that synthesize vast amounts of evidence to facilitate clinical decision-making for busy HCPs who struggle to keep pace with the rapid dissemination of new findings (13). However, the credibility of CPGs, including the relevance, accuracy, and representativeness of the evidence used, is rarely evaluated, despite criteria for trustworthy CPGs being developed by the Institute of Medicine (IOM) (12). A review of CPGs focusing on pediatric populations found that only 75% of 216 included CPGs were evidence-based (14). Similar reviews in other healthcare domains have shown that some recommendations lack supporting evidence or inflated the strength of recommendations compared to the supporting evidence (15, 16). This highlights the need to critically evaluate the quality of CPGs and the underlying evidence, as these recommendations will influence clinicians' decision-making and patient care.

Traditionally, CPGs for pain, sedation, delirium, and IWS management in pediatric intensive care have focused on a sole condition or two. However, there is a growing emphasis for a more integrated approach to managing these four conditions (8, 17). Existing systematic reviews of CPGs related to either of the four conditions have primarily focused on pain and not specifically related to PICU care, e.g., procedural pain in neonates (18), or acute pain in burn patients (19). To date, no systematic evaluation has been undertaken to assess the quality of CPGs and their recommendations for the management of pain, sedation, delirium, and IWS in pediatric intensive care. This systematic review aims to identify and assess the quality of CPGs, focusing on the management of these four conditions. The objectives include evaluating the quality of recommendations, synthesizing recommendations, harmonizing the strength of the recommendation (SoR) and the certainty of evidence (CoE), and assessing the accuracy and relevance of supporting evidence.

## 2. Methods

This review followed the methodological guide for conducting systematic reviews of CPGs (20) and used the preferred reporting items for systematic review and meta-analysis (PRISMA) for reporting (21). A study protocol was published prior to conducting the review (22), and is registered in the international database of prospectively registered systematic reviews (PROSPERO ID CRD42021274364).

### 2.1. Inclusion/exclusion criteria for study selection

The eligibility criteria for selecting CPGs were predetermined using the population, intervention, comparators, attributes, and recommendations (PICAR) framework (20). In this case, the population of interest was children (newborn to 18 years of age), and the intervention was the management of one of the four conditions. The comparator were CPGs with children-specific recommendations that could be implemented in a PICU (Supplementary Table S1). The attributes and recommendations were included in the eligibility criteria, which were the CPGs must: (i) contain at least one recommendation for assessing any of the four conditions, (ii) be applicable to the PICU setting, (iii) be endorsed by a professional society, and (iv) be the most current version. The publication year was limited to January 1, 2010-May 30, 2023, with no language restrictions. CPGs focusing on specific types of procedures or surgeries, and neuromuscular blockade were excluded to provide a general overview of managing the four conditions (22) (please refer to published protocol for more details).

### 2.2. Search methods

#### 2.2.1. Information sources

To identify eligible CPGs, a search was conducted on January 4, 2022, using the following information sources:

(1) Four electronic databases: Medline ALL (Ovid), Embase.com, CINAHL with Full Text (EBSCO), and JBI EBP Database (Ovid). Updated on May 26, 2023.
(2) Nine guideline repositories.
(3) Thirteen professional society websites. Updated on May 26, 2023.
(4) Forward citation searches using Google Scholar and society websites were performed to find the most current version of each CPG. Updated on May 26, 2023.
(5) Experts in the field, which was added as an additional source following the publication of the protocol.

#### 2.2.2. Search strategy

The search strategy adapted to each information source was developed with the assistance of a health information specialist (AT), using index and free-terms describing the concepts of: (1) pain, sedation, delirium, and IWS, and (2) CPGs. The search strategy was peer reviewed by another librarian, following the PRESS checklist (23). The full search strategies and details are available in Supplementary Tables S2–S4.

### 2.3. Guideline selection

Retrieved records were imported into Endnote 20 reference manager (Clarivate Analytics, USA) and duplicates removed (AT). Screening and full-text review processes were performed by two independent reviewers (IMD and SA) using Rayyan QCRI (Qatar Computing Research Institute, Doha, Qatar) (24). Disagreements were resolved through consensus.

### 2.4. Data collection and translation process

A search for supplementary materials for included CPGs was conducted, corresponding authors were contacted, when necessary, but no additional information was obtained. CPGs published in languages other than English or French were translated using standardized translation methods (25). An initial translation was performed using Deepl (26) and the document was sent to a volunteer translator who was both a content expert and a native speaker of the original language of the included CPG [MM: German, EI: Dutch (acknowledged), YG: Chinese] for proofreading, editing and verification.

### 2.5. Data extraction and synthesis

One reviewer (IMD) extracted information from each included CPGs and was independently verified by a second (SA). A predefined data extraction Excel form was developed and pilot tested. Key areas of extracted data included: (i) general information about CPGs; (ii) Appraisal of Guidelines for Research and Evaluation (AGREE II) quality appraisal (27) (details provided below); (iii) AGREE Recommendation Excellence (AGREE-REX) to assess the quality of recommendations for medium and above quality CPGs (28) (details provided below); and (iv) recommendations from each CPG categorized by the four conditions and type of recommendation.

#### 2.5.1. Quality appraisal of CPGs and recommendations

The AGREE II, a validated appraisal instrument was designed to evaluate the quality of CPGs (27). It contains 23 items across six domains: (1) scope and purpose, (2) stakeholder involvement, (3) rigour of development, (4) clarity of presentation, (5) applicability, and (6) editorial independence. Each item is rated on a 7-point Likert scale ranging from 1 (strongly disagree) to 7 (strongly agree). The AGREE II also includes two global rating scores: (1) one used to assess the overall quality of the CPG (rated on the 7-point Likert scale), and (2) another to indicate whether the guideline would be recommended for use (rated as either yes, yes with modifications, or no).

The AGREE-REX instrument was used to assesses the quality of the CPG recommendations (28). It contains nine items across three domains: (1) clinical applicability; (2) values and preferences; and (3) implementability. Each item is appraised using a 7-point Likert scale ranging from 1 (strongly disagree) to 7 (strongly agree). The AGREE-REX includes two global rating scores: (1) one to assess the overall quality of the CPG recommendations, and (2) one for recommended use in a specific context (this rating was used based on the applicability to the PICU setting).

The score for the AGREE II is determined by summing the scores across all reviewers and converting them to a percentage of the maximum possible score for each domain. All authors contributed towards appraisal, with three independently evaluating each CPG, with one reviewer responsible for appraising all CPGs (IMD).

In order to categorize the quality of CPGs using the AGREE II, domains scores were classified into three categories based on thresholds: high-quality (≥60%), medium-quality (scores between 30% and 60%), and low-quality (<30%) (29). In the protocol, all domains had to be used to determine quality classification. However, a deliberate deviation was made by applying the quality criterion exclusively to domain 3: rigor of development. This decision was based on the inclusion of all types of guidance documents and that even rigorously developed CPGs can fall short on the other domains.

Following the AGREE II appraisal, CPGs that met the threshold for medium- and high-quality levels proceeded to the quality appraisal of recommendations stage using the AGREE-REX. An additional criterion was added: if at least two appraisers indicated that they would not recommend the use of the CPG, it did not proceed further. This applied to only one CPG, which had a borderline quality threshold of medium-quality in domain 3. A consensus meeting was held for each CPG with at least two reviewers scoring each item in the AGREE-REX. AGREE-REX scores were converted to a percentage in the same manner as the AGREE II.

Since training tools for the AGREE II were not available at the time of the review (www.agreetrust.com), the review team developed training videos on the AGREE II and selected a sample CPG for training purposes to ensure inter-rater reliability. Each reviewer watched the videos, completed the sample guideline, and met with the review lead (IMD) to discuss results before appraisal of assigned CPGs. Inter-rater agreement was calculated in SPSS version 27 using intra-class correlation coefficients (ICCs) and a two-way random, absolute agreement model for all AGREE II scores between the three raters. The level of ICC agreement was considered poor (<0.50), moderate (0.50–0.75), good (0.75–0.9), or excellent (>0.9) (30).

The quality scores for each domain in the AGREE II and AGREE-REX are presented as a heat map using the previously described quality thresholds.

#### 2.5.2. Recommendation synthesis

Child-specific recommendations were extracted from CPGs rated as medium-quality or above. Recommendations specific to the management of the four conditions were extracted, while those relating to the perioperative period, neuromuscular blockade, short-term procedures, or postoperative management of specific types of surgeries were excluded. Each recommendation was extracted and categorized per the four conditions, and the SoR, CoE, and supporting references were recorded into an Excel spreadsheet. The recommendation synthesis process consisted of three-steps.

1. Categorization: All recommendations were categorized into five categories: (1) assessment, (2) management, (3) implementation (4) education, and (5) organizational/policy. Recommendations could belong to multiple categories. Details on the categories and sub-categories and their modifications compared to the protocol are found in Supplementary Table S5.
2. Review by condition and category: By condition each category was reviewed (e.g., all pain recommendations categorized under assessment), and using thematic analysis, similar underlying management recommendations found in at least two CPGs were combined to create a summary recommendation.
3. Comparison of all summary recommendations: All summary recommendations were compared to each other, if similar recommendations existed across multiple conditions, they were combined into a single summary recommendation. For example, a summary recommendation for a pain protocol and a summary recommendation for a sedation protocol that both included analgosedation were combined.

#### 2.5.3. Harmonization of the SoR and CoE for summary recommendations

The SoR and CoE from each original recommendation were harmonized to facilitate comparison and interpretation across the medium-quality and above CPGs. This harmonization process involved creating two tables (one for SoR and one for CoE) based on the systems used in each CPG, following the method described by Krugar et al. (31). These tables, along with a detailed description of the harmanization process, can be found under results section 3.4 synthesis of recommendations.

Overall SoR for each summary recommendation was established based on adapted criteria from Corp et al. (32), and were categorized as “*Strong*”, “*Conditional*”, “*Inconsistent*”, “*Inconclusive*”, and “*Good practice*”.

Overall CoE for each summary recommendation were categorized as: “*High*”, “*Moderate*”, “*Low*”, “*Very low*”, “*Inconsistent*”, “*Inconclusive*” and “*Conditional*”. The outcome of this process is a final table presenting the summary recommendations with the harmonized SoR and CoE.

#### 2.5.4. Review of supporting evidence

The review of supporting evidence involved one reviewer (IMD), who evaluated the relatedness of the cited literature to each recommendation (yes, no, mixed) and determined the level of support (fully, partially, not at all) for each recommendation.

## 3. Results

### 3.1. Study selection

**Figure 1:**
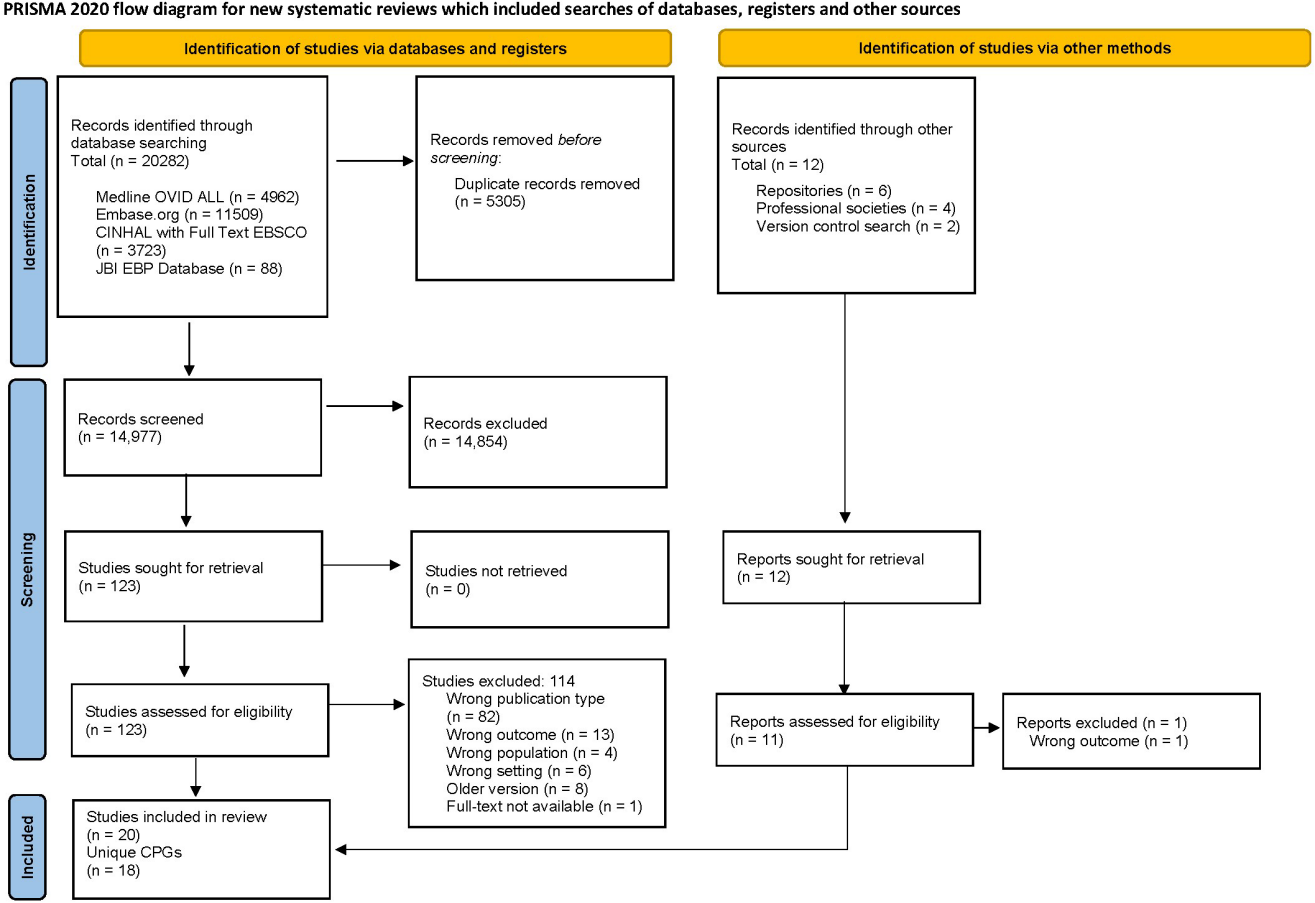
The PRISMA flow diagram summarizes the number of studies excluded in each phase of the selection process (21).

A total of 14,977 records were identified from the electronic databases after removing duplicates. Of these, 123 studies underwent full-text review, and 9 articles met the eligibility criteria. Another 11 articles were identified through the guideline repositories and society website searches. In total, 20 articles, representing 18 unique CPGs, were identified (8, 33–51). For a detailed overview of the selection process, see the PRISMA flow diagram (21) in Figure 1. Additional information on the excluded studies and reasons for exclusion are found in Supplementary Tables S6.

### 3.2. Characteristics of CPGs and development process

#### 3.2.1. Characteristics of CPGs

The main characteristics of the included CPGs are summarized in Table 1. Among the included CPGs, more than half were published in European countries (55.5%, *n* = 10) (8, 33–43), while five originated from North America (28%) (44–48), two from Asia (11%) (49, 50), and one from Australia (5.5%) (51). The description of the guidance document types varied among the included CPGs: nine were categorized as guidelines (CPG or guideline) (50%) (35, 39–46), three were classified as recommendations (recommendation or clinical practice recommendation) (16.7%) (33, 34, 46), three were consensus documents (consensus recommendations or expert consensus) (16.7%) (36–38, 49, 50), and one each fell into the categories of position statement (8), book (51), or practice alert (48) (5.5% each).

**Table 1:** Characteristics of the included Clinical practice guidelines.

Society, year (ref)	Title	Country/region	Language	Type of CPG	Version	Population	Setting	Condition of focus				System for Certainty of evidence (cat)	System for strength of recommen-dation (cat)	Patient on panel	Used AGREE II
								P	S	D	W				
Dutch Society of Anaesthesiology (NVA), 2012 (42)	Guideline postoperative pain	Netherlands	Dutch	Guideline	Updated from 2003	A, C, NC	G (postoperative wards)	▪	□	□		EBRO (A1, A2, B, C, D)	NI	Y	Y
Nederlands Vereniging voor Psychiatrie (NVvP), 2014 (41)	Multidisciplinary guideline paediatric delirium	Netherlands	Dutch	Guideline	Adapted (nvic, 2010) (nvKg, 2013)	C, NC	S (PICU), G (ER, medium wards, hospital)	□	□	▪	□	EBRO (A1, A2, B, C, D)	GRADE (Level 2,3,4) ^b^	N	Y
Registered Nurses’ Association of Ontario (RNAO), 2013 (45)	Assessment and management of pain	Canada	English	Guideline	Updated from 2007 (52)	A, C, NC	ALL	▪	□			SIGN^a^ (Ia, Ib, IIa, IIb, III, IV)	NI	N	Y
French Society of Anaesthesia and Intensive Care Medicine (SFAR), 2019 (35)	Revision of expert panel's guidelines on postoperative pain management	France	English	Guideline	Updated from 2008 (53)	C, NC	G (postoperative wards)	▪				GRADE (high, moderate, low, very low)	GRADE (strong (1+/1-), weak (2+/2-))	N	N
European Society for Paediatric and Neonatal Intensive Care (ESPNIC), 2016 (8)	Clinical recommendations for pain, sedation, withdrawal and delirium assessment in critically ill infants and children: an ESPNIC position statement for healthcare professionals	Europe	English	Position statement	Original	C, NC	S (PICU)	▪	▪	▪	▪	CBO (1+, 1-, 2, 3, 4) ^b^	CBO (A, B, C, D) ^b^	N	N
Society of Critical Care Medicine (SCCM), 2022 (44)	2022 Society of Critical Care Medicine Clinical practice guidelines on prevention and management of pain, agitation, neuromuscular blockade, and delirium in critically Ill pediatric patients with consideration of the ICU environment and early mobility	USA	English	Clinical practice guideline	Original	C, NC	S (PICU)	▪	▪	▪	▪	GRADE (high, moderate, low, very low)	GRADE (strong, conditional, good practice)	N	N
Italian Society of Neonatal and Pediatric Anesthesia and Intensive Care (SARNePI), 2014 (34)	Recommendations for analgesia and sedation in critically ill children admitted to intensive care unit	Italy	English	Recommendations	Original	C	S (PICU)	▪	▪	▪	▪	GRADE (strong, moderate, weak)	GRADE (strong = we recommend, moderate or weak = we suggest)	N	Y
SARNePI, 2022 (33)	Update of recommendations for analgosedation in pediatric intensive care unit	Italy	English	Recommendations	Original	C, NC	S (PICU admitting neonatal population)	▪	▪	▪	▪	SIGN (level 1++, 1+, 1-, 2++, 2+, 2-, 3, 4)	SIGN (A, B, C, D)	N	Y
Australian and New Zealand College of Anaesthetists and Faculty of Pain Medicine (ANZCA & FPM), 2020 (51)	Acute pain management: Scientific evidence	Australia	English	Book	Updated from 2015 (54)	A, C, NC	G	▪				NHMRC (I, II, III-1, III-2, III-3, IV, expert opinion)	Johnson et al., 2003—method for updating recommendations (new, unchanged, strengthened, weakened, qualified, reversed, NB)	N	N
Saudi Society of Pain Medicine (SSPM), 2021 (49)	Postoperative pain management in Saudi Arabia: Consensus recommendations from a Saudi Expert Panel	Saudi Arabia	English	Consensus recommendations	Adapted from ANZCA (54), SAFR (35), APS & ASA (46), PROSPECT (55)	A, C	G	▪	□			NI (low, moderate, high)	NI (weak, strong)	N	N
American Society for Pain Management Nursing (ASPMN), 2019 (47)	Pain assessment in the patient unable to self-report: Clinical practice recommendations in support of the ASPMN 2019 Position Statement	USA	English	clinical practice recommendations	Updated from 2011 (56)	A, C, NC	G	▪				NI	NI	N	N
American Pain Society, the American Society of Regional Anesthesia and Pain Medicine, and the American Society of Anesthesiologists (APS & ASA), 2016 (46)	Management of postoperative pain: A clinical practice guideline from the American Pain Society, the American Society of Regional Anesthesia and Pain Medicine, and the American Society of Anesthesiologists’ Committee on Regional Anesthesia, Executive Committee, and Administrative Council	USA	English	Clinical practice guideline	Original	A, C, NC	All	▪	□		□	GRADE^a^ (high, moderate, low)	GRADE^a^ (weak or strong)	N	N
Association of Paediatric Anaesthetists of Great Britain and Ireland (APA), 2012 (40)	Good practice in postoperative and procedural pain management	UK, Ireland	English	Guideline	Updated from 2008 (57)	C, NC	G (hospital)	▪				NI (1 (1++,1+, 1-),2 (2++, 2+,2-),3 (case report/case series),4 (expert opinion))	SIGN (A,B,C,D)	Y	N
Chinese Medical Association (CMA), 2018 (50)	Experts’ consensus on sedation and analgesia for children in pediatric intensive care unit of China	China	Chinese	Expert consensus	Updated from 2013	C, NC	S (PICU)	▪	▪	□	□	NI	NI	N	N
Austrian Society for Anesthesiology, Resuscitation and Intensive Care Medicine (ÖGARI), 2014 (36–38)	Austrian interdisciplinary recommendations for action for perioperative pain management in children	Austria	German	Consensus recommendations	Adapted- from DAS 2009 and ANZCA 2010 (58)	C, NC	G (neonat and pediatric postoperative wards)	▪				ÖGARI system “based on” CEBM (A (=1); B (=2–3); C(=4–5))	ÖGARI system (A^a^, A, B, C)	N	Y
American Association of Critical-Care Nurses (AACN), 2016 (48)	AACN practice alert: assessment and management of delirium across the lifespan	USA	English	Practice alert	Original	A, C, NC	NI (include ICU & PICU but no indication)			▪		AACN levels (A, B, C, D, E, M)^b^	NI	N	N
Arbeitsgemeinschaft der Wissenschaftlichen Medizinischen Fachgesellschaften, (AWMF—the Association of the Scientific Medical Societies), 2020 (39)	Analgesia, sedation and delirium management in intensive care medicine S3 guideline	Germany	German	Guideline	Updated from 2015 (59)	A, C, NC	S (ICU, PICU)	▪	▪	▪	□	OCEBM (1a, 1b, 1c; 2a, 2b, 2c; 3a, 3b, 4; 5, +++, ++++, ++, 1, expert consensus)	AWMF system of voting (A (strong recommendation), B (recommendation), O (open recommendation))	Y	N (Y for 2015 version)
Polish Society of Anaesthesiology and Intensive Therapy (PSAIT), 2022 (43)	Guidelines for treatment of acute pain in children—the consensus statement of the Section of Paediatric Anaesthesiology and Intensive Therapy of the Polish Society of Anaesthesiology and Intensive Therapy	Poland	English	Guideline	Original	C, NC	NI (includes NICU and PICU)	▪				NI (I, II, IIa, IIb, III)	NI (Level A, B, C)	N	N

▪ = Recommendation, □ = Included in body of evidence or discussion, NI = Not included or indicated.

**Population**: A, adult; C, child; NC, non-communicative. **Setting**: G, general; S, specific; PICU, pediatric intensive care unit; ER, emergency department; ALL also includes setting outside of acute care/hospital settings (i.e. community). Cat, categpry. **Level of evidence grading**: CBO, Dutch Institute for Healthcare Improvement; GRADE, Grading of Recommendations Assessment, Development and Evaluation; EBRO, Evidence Based Recommendation Development; SIGN, Scottish Intercollegiate Guidelines Network; OCEBM, Oxford Center for Evidence-based Medicine; NHMRC, National Health and Medical Research Council; PROSPECT, PROcedure-SPECific Pain ManagemenT.

^a^ Adapted.

^b^ No explantation or description was given on the interpretation of levels of evidence or grade of recommendation.

Regarding the nature of development of the 18 CPGs, seven were original publications (39%) (8, 33, 34, 43, 44, 46, 48), eight were updates of previously published CPGs (44%) (35, 39–41, 45, 47, 50, 51) and three were adapted from other CPGs (17%) (36–38, 42, 49). Five CPGs were translated from other languages (28%) (36–39, 41, 42, 50), while the remaining were available in English (72%) (8, 33–35, 40, 43–49, 51). Eight CPGs had a target population that included both adult and pediatric populations (44%) (39, 42, 45–49, 51). The remaining 10 were specifically developed for pediatrics (56%) (8, 33–38, 40, 41, 43, 44, 50).

The focus of conditions (pain, sedation, delirium, and IWS) in the CPGs varied. Pain was the focus in 10 CPGs (56%), with six solely addressing pain (60%) (35–38, 40, 43, 47, 51), and four including other conditions indirectly within the body of evidence (40%) (42, 45, 46, 49). Two CPGs focused on delirium (11%), with one solely addressing it (48), and the other including other conditions indirectly (41). One CPG addressed pain and sedation (5.5%) (50). Five CPGs covered all four conditions (28%), with four directly including all (80%) (8, 33, 34, 44), and one indirectly including IWS (20%) (39).

#### 3.2.2. Characteristics of key CPG development processes

The majority of CPGs utilized a multi-disciplinary panel for their development (89%, *n* = 16) (8, 33–42, 44–48, 50, 51), while two CPGs did not report the development process at all (11%) (43, 49). Among the included CPGs, four development groups mentioned including patients as representatives on the CPG panel (22%) (39–42), although the amount of their involvement was not described in detail. In two CPGs development groups used additional methods to gather patient and family experiences (11%), one used a survey (33), and another used parent interviews (41).

For the evaluation of the SoRs, eight CPGs used one of three formal systems (56%) (8, 33–35, 41, 44, 46, 51). The most used was Grading of Recommendations Assessment, Development and Evaluation (GRADE) by five CPGs (28%) (33, 35, 41, 44, 46), followed by Scottish Intercollegiate Guidelines Network (SIGN) used by two (11%) CPGs (34, 40), and Dutch Institute for Healthcare Improvement (CBO) used by one CPG (5%) (8). Three CPGs used their own developed or adapted systems for SoRs (17%) (36–39, 51), and seven CPGs did not provide information on the system used for SoR evaluation (39%) (42, 43, 45, 47–50).

For evaluating the CoE, eleven CPGs used one of six formal systems (56%) (8, 33–35, 39, 41, 42, 44–46, 51). The most used system was GRADE (*n* = 4, 22%) (33, 35, 44, 46), followed by SIGN (34, 45) and Evidence Based Recommendation Development (EBRO) (41, 42), used by two CPGs each (11%). One CPG each used the National Health and Medical Research Council (NHMRC) (5.5%) (51), CBO (5.5%) (8), and Oxford Center for Evidence-based Medicine (OCEMB) (5.5%) (39). The Austrian grouped CPG used an adapted CoE system (5.5%) (36–38), while the American Association of Critical-Care Nurses (AACN) used their own system for assessing the CoE (5.5%) (48). Five CPGs did not report the system used for assessing the CoE (28%) (40, 43, 47, 49, 50).

In terms of using the AGREE II for assisting with development or reporting quality, six CPGs reported using it during either of these stages (33%) (33, 34, 36–38, 41, 42, 45). Regarding revising and updating, 10 CPGs (56%) provided a timeframe for this process. Four CPGs were recently published, therefore not requiring an update (33, 39, 44, 51), and among the remaining six CPGs, one has a planned revision for next year but is still overdue (45), and the others have exceeded the indicated timeframe for updating without having completed the process (36–38, 40–42, 46).

### 3.3. Quality appraisal

#### 3.3.1. AGREE II quality appraisal of CPGs

The results of the overall AGREE II domains appraisal are displayed in Table 2. Three CPGs rated as high-quality (33, 39, 45), nine as medium-quality (8, 35–38, 40–42, 44, 46, 51), and six as low-quality (34, 43, 47–50). The highest mean scores were for Domain 1: Scope and purpose and Domain 4: Clarity of presentation (both 66%). The lowest mean score was for Domain 5: Applicability (21%). The lowest mean score per item (<2) was for item 5: The views and preferences of the target population have been sought (1.8), and item 20: The potential resource implications of applying the recommendation have been considered (1.7). Another five items had a mean of less than 3 (items: 8, 9, 13, 18, 21). The highest mean score item was item 1: The overall objective(s) of the guideline is (are) specifically described (5.6), followed by item 17: Key recommendations are easily identifiable (5.2). Raw scores for individual items of the AGREE II, for the three reviewers for each CPG is available in Supplementary Table S7. Inter-rater reliability varied, with two CPGs rated as poor (<0.50) (40, 43), six as moderate (0.50–0.75) (8, 35, 41, 42, 45, 50), 10 as good (0.75–0.9) (33, 34, 36–39, 44, 46–49, 51), and none as excellent (>0.9).

**Table 2:** Heatmap of CPG AGREE II, AGREE-REX and inter-rater agreement.

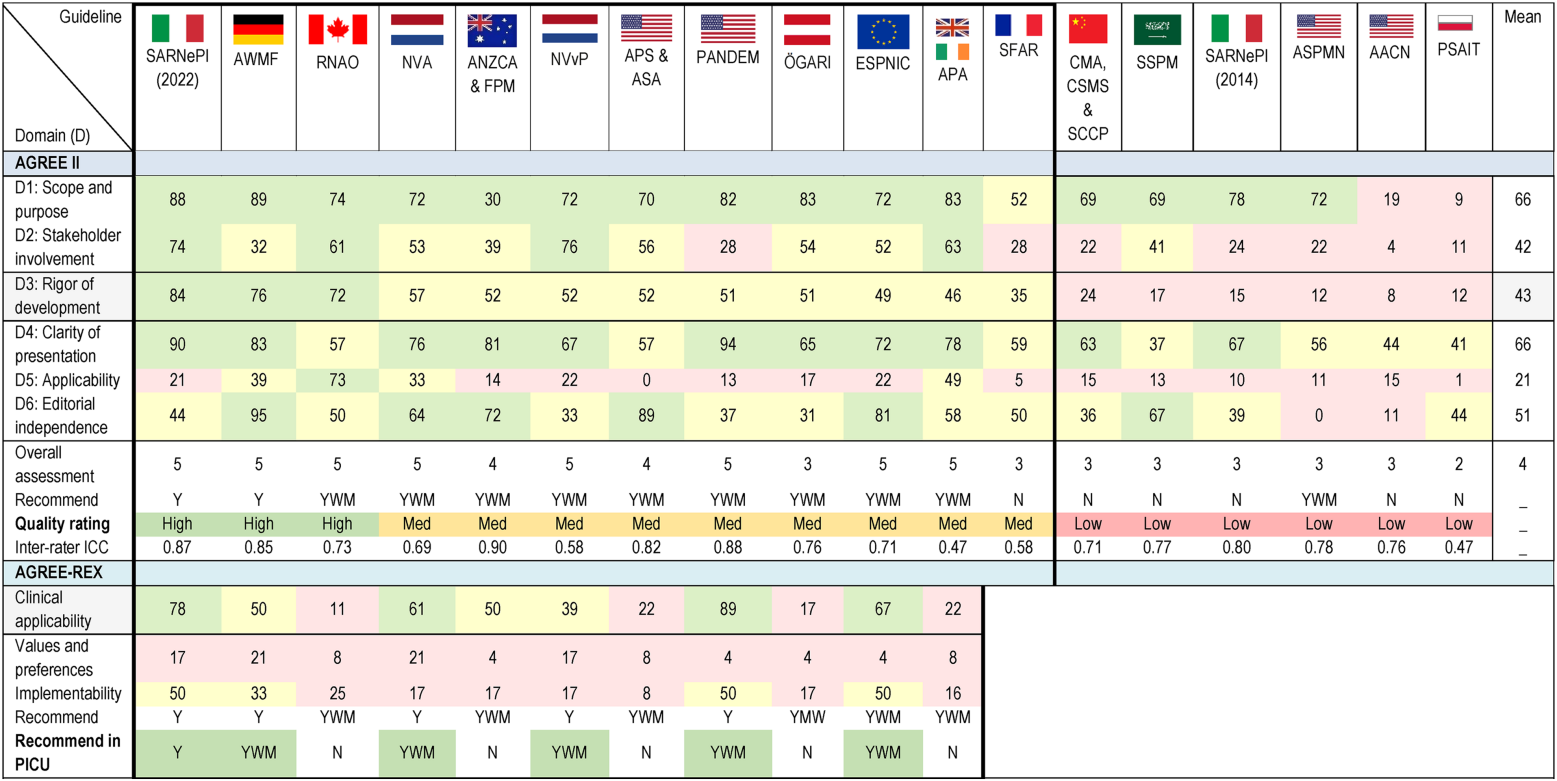

Note: The degree of reviewer score agreement was defined using a previously used scale: <0.20 = poor; 0.21-0.40 = fair, 0.41-0.60 = moderate, 0.61-0.80 = good, 0.81-1.00 = very good, ICC = intraclass correlation coefficient.

Threshold colors: ▪ = critical domain towards threshold determination; Thresholds = ▪ High ▪ Medium ▪ Low; Med = Medium; Y = Yes; YWM = Yes with modifications; N = No.

Abbreviations: AACN, American Association of Critical-care Nurses; ANZCA & FPM, Australian and New Zealand College of Anaesthetists and Faculty of Pain Medicine; APA, Association of Paediatric Anaesthetists; APS & ASA, American Pain Society & the American Society of Anesthesiologists; ASPMN, American Society for Pain Management Nursing; AWMF, the Association of the Scientific Medical Societies; CMA, CSMS & SCCP, Chinese Medical Association, Chinese Society of Medical Science & Society of Critical Care Physicians; ESPNIC, European Society of Paediatric and Neonatal Intensive Care; NVA, Dutch Society of Anaesthesiology; NVvP, Nederlands Vereniging voor Psychiatrie; ÖGARI, Austrian Society for Anesthesiology, Resuscitation and Intensive Care Medicine; PSAIT, Polish Society of Anaesthesiology and Intensive Therapy; RNAO, Registered Nurses' Association of Ontario; SARNePI, Italian Society of Neonatal and Pediatric Anesthesia and Intensive Care; SFAR, French Society of Anaesthesia and Intensive Care Medicine; SSPM, Saudi Society of Pain Medicine.

**Figure 2:**
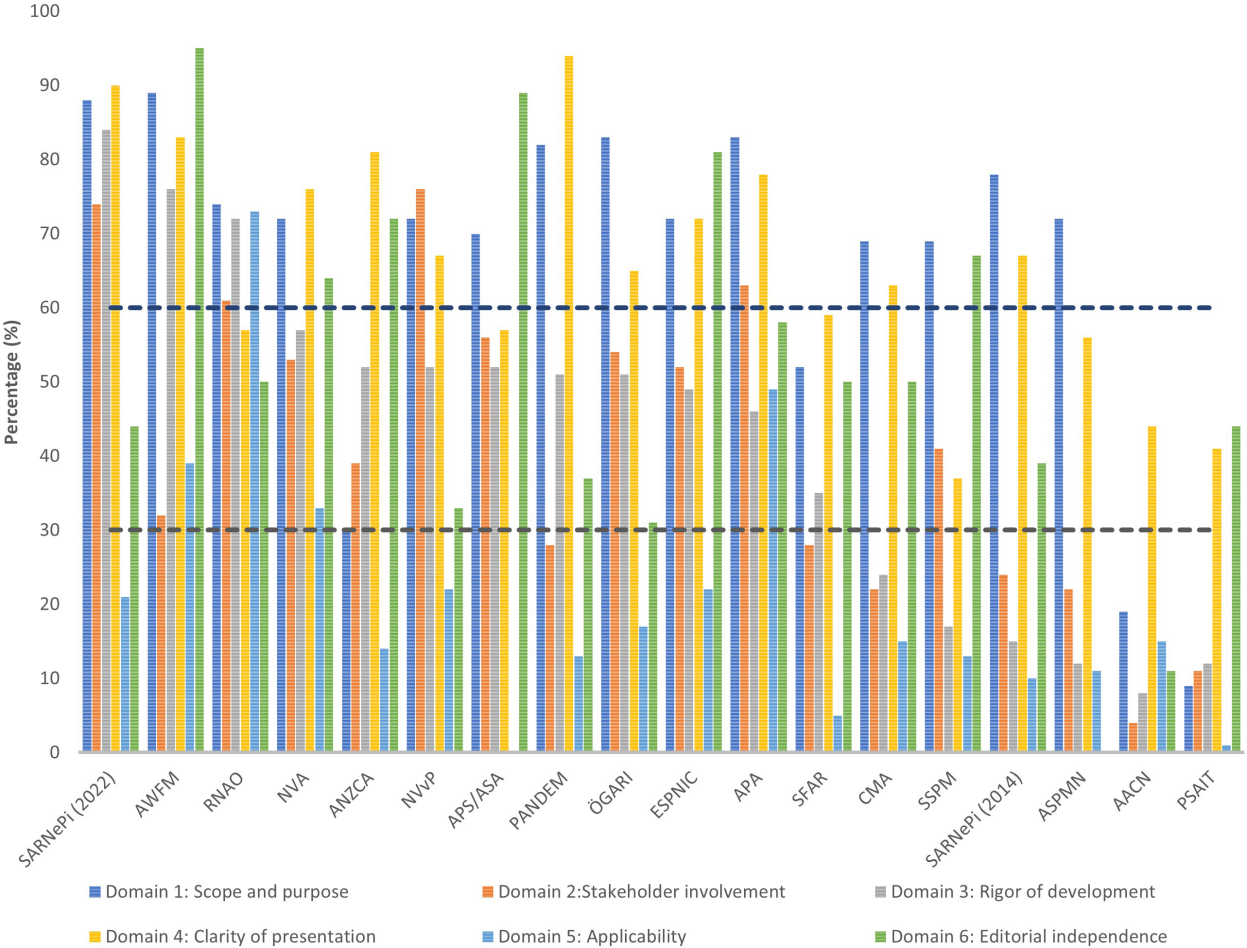
The AGREE II scores are displayed by each domain across all CPGs. The dashed lines represent the cut-off thresholds: low = >30%, medium = 30%–59%, and high = <60%.

The overall AGREE II results are displayed in Figure 2. This shows that for four CPGs all domains scored above the lowest threshold (<30%) (39, 40, 42, 45).

#### 3.3.2. Quality appraisal of recommendations

Eleven CPGs were included in the AGREE-REX appraisal (8, 33, 36–42, 44–46, 51), the details of the REX consensus meeting are presented in Table 2. Four CPGs scored as high-quality based on Domain 1 (8, 33, 42, 44). Overall domain scores ranged from 4% to 89%. In order from highest mean score to lowest was Domain 1: Clinical applicability (46%), Domain 3: Implementability (27%), and Domain 2: Values and preferences (11%). The highest mean score for individual items was for item 2: Applicability to target users (4.6), one item, item 7: Values and preferences of guideline developers received no score. Six out of the nine items had a mean score of <3 (items: 3, 4, 5, 6, 7, 9). All eleven CPGs were recommended for use in the appropriate context. For use in the PICU, one CPG was recommended (33), five were recommended with modifications (8, 39, 41, 42, 44), and five were not recommended (36–38, 40, 45, 46, 51).

### 3.4. Synthesis of recommendations

A total of 170 recommendations were extracted from the six medium-quality and above CPGs recommended for use in the PICU (8, 33, 39, 41, 42, 44). All recommendations and inclusion/exclusion decisions can be found in Supplementary Tables S8. These recommendations were categorized by condition, resulting in 65 recommendations for pain, 40 for sedation (14 of which were repeated under other conditions due to overlapping conditions within recommendations), 61 for delirium (eight repeated under other conditions), 20 for IWS (four repeated under other conditions), and 13 organizational recommendations (three repeated under other conditions). During the grouping process, 77 recommendations could not be grouped and were excluded from further synthesis. As a result of the process for recommendation grouping, 30 summary recommendations were created which are presented in Table 3. An example of the additional details for the summary recommendations for pain assessment, including the review of consistency across CPGs for SoR, and CoE, and the review of evidence including relevance and support, is provided in Supplementary Table S9 (the complete file is available on request).

**Table 3:** Summary of recommendations across six CPGS for pain, sedation, delirium, IWS.

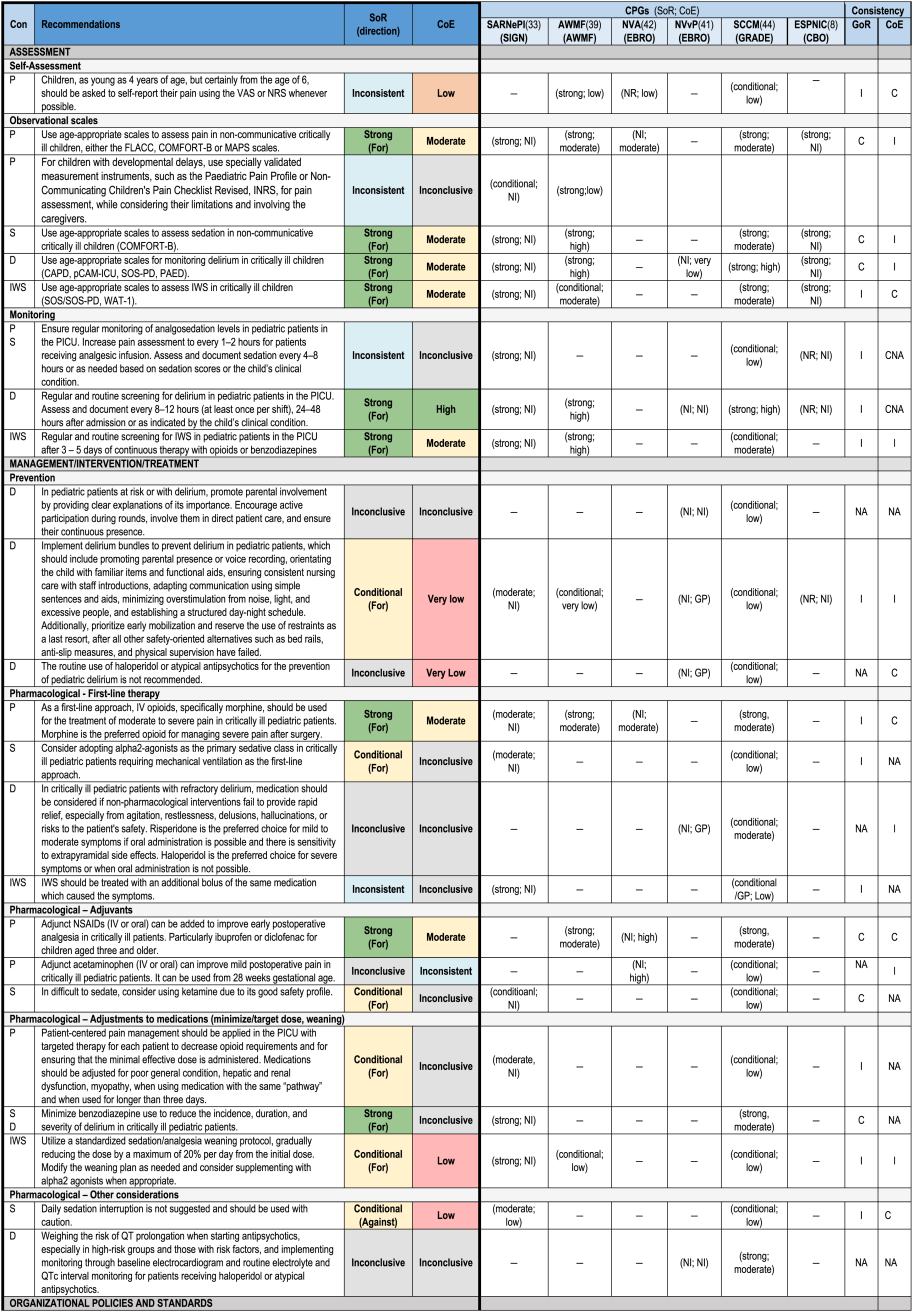
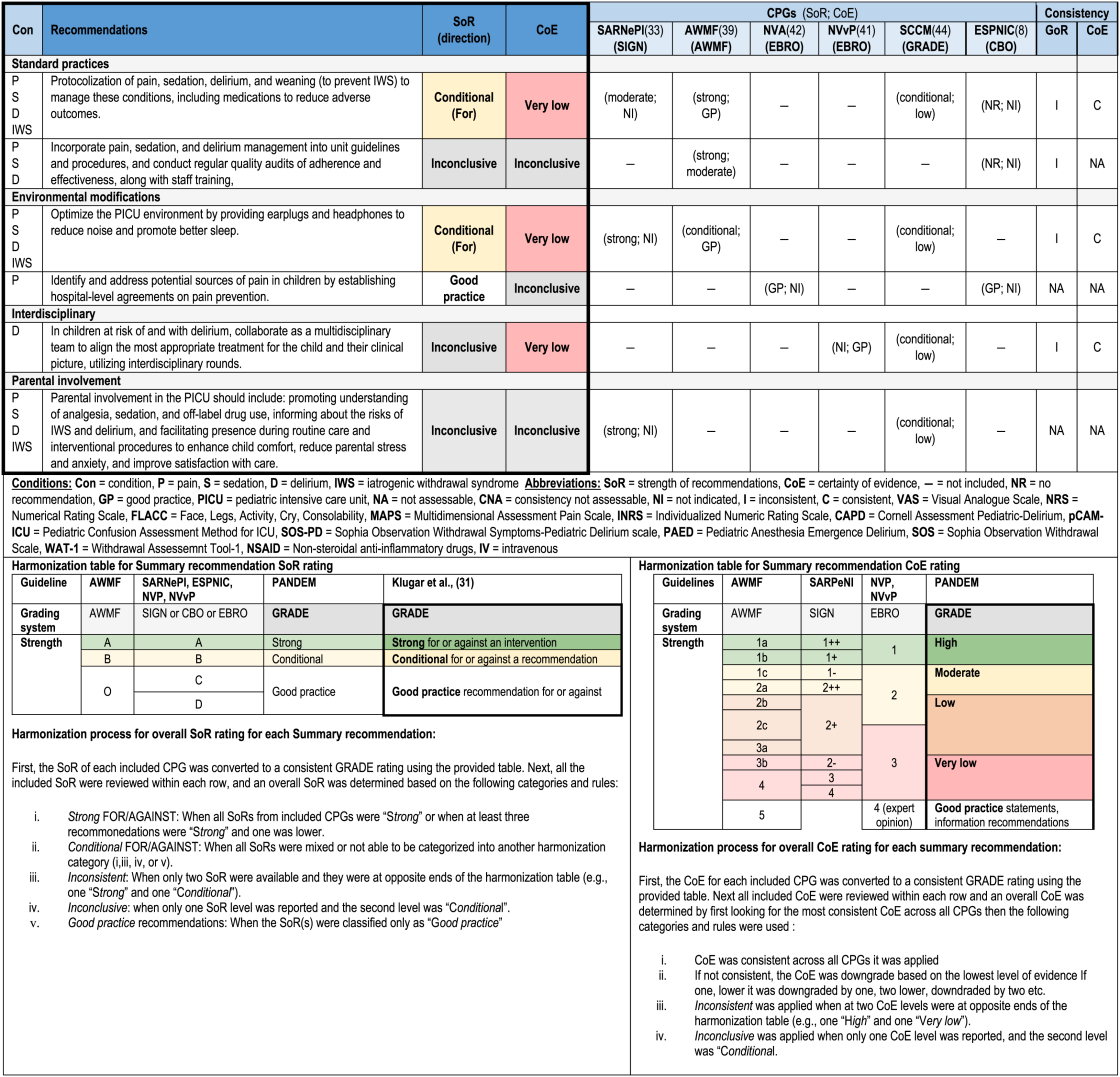

#### 3.4.1. Pain

A total of 13 summary recommendations were specifically related to pain. Among these, seven were specific to pain management, and five addressed a combination of other conditions, including pain (these will be described separately). These pain specific recommendations included three on assessment, one each on self-assessment, observational scales, and routine screening. There were four recommendations on pharmacological management of pain. The level of consistency between summary recommendations for SoR and the CoE varied, in that the supporting evidence for the recommendations on observational scales and medications were strong, while the evidence for routine screening intervals lacked evidence-based support.

#### 3.4.2. Sedation

A total of 10 summary recommendations were specifically related to sedation. Among these, five were specific to sedation management, and five addressed a combination of other conditions, including sedation (described separately). The sedation specific recommendations included one on assessment using observational scales, one on monitoring, two on pharmacological management, and one on another management approach which is a recommendation against daily sedation interruption. The level of consistency between the included grouped recommendations for SoR and the CoE varied.

#### 3.4.3. Delirium

A total of 13 summary recommendations were specifically related to delirium. Among these, eight summary recommendations were specific to delirium management, and five recommendations addressed a combination of other conditions, including delirium (described separately). The delirium specific recommendations included one on assessment using observational scales, one on monitoring, three on prevention, two on pharmacological management, and one on another management approach. The level of consistency between the included grouped recommendations for SoR and the CoE varied, in that the recommendations related to assessment and monitoring were consistent; however, the remaining recommendations were inconclusive, and many lacked supporting evidence.

#### 3.4.4. Iatrogenic withdrawal syndrome

A total of seven summary recommendations were specifically related to IWS. Among these, four summary recommendations were specific to IWS management, and three recommendations addressed a combination of other conditions, including IWS (described separately). The IWS specific recommendations included one on assessment using observational scales, one on monitoring, and two on pharmacological management, with one of these being specific to weaning of medications. The level of consistency between the included grouped recommendations for SoR and the CoE varied.

#### 3.4.5. Other—organizational/policy

Five summary recommendations addressed organizational factors. Among these, one focused on monitoring of analgosedation (pain and sedation), another was on the implementation of policies and procedures (pain, sedation, and delirium), and the remaining three recommendations included all four conditions for the use of protocols/algorithms to standardize management, modifications to the PICU environment, and involvement of parents. There was inconsistency between SoR and CoE for all these organizational recommendations, with minimal supporting evidence.

## 4. Discussion

This systematic review, to the best of our knowledge, is the first comprehensive assessment of CPGs for the management of pain, sedation, delirium, and IWS in PICU. It is also unique in its approach to incorporating and evaluating these four conditions together, which has emerged as a novel approach in the field (60). Through an extensive search, 18 CPGs and 170 child-specific recommendations were identified from medium- and high-quality CPGs, which were synthesized into 30 summary recommendations. One of the key findings is that most of the identified CPGs were medium-quality, with only a small percentage categorized as high-quality (17%). The synthesized summary recommendations covered various aspects of care, including pharmacological management (*n* = 12, 41%), assessment and monitoring (*n* = 8, 28%), and organizational policy (*n* = 6, 21%) approaches. Notably, CPGs focused on delirium were lacking, which is consistent with current clinical practice surveys (9–11). Additionally, a recent review of IWS implementation strategies found similarities to the IWS summary recommendations (61).

Although, the evidence base is mostly inconsistent and includes small number of studies, the summary recommendations presented provide the best available evidence for managing critically ill children at risk for under-treated pain, over-sedation, and the consequences of delirium and IWS and can serve as a valuable resources for HCPs in the PICU. However, implementing recommendations from included CPGs requires attention, as many lacked information or resources for implementation, supported by the very low scores in the AGREE II domain of applicability. This is an issue commonly identified in other pediatric systematic reviews utilizing the AGREE II instrument (18, 62). The implementation of protocolized approaches for pain, sedation, and delirium was a common recommendation across the included CPGs, although, the harmonized SoR and CoE was conditional and low. Our recent systematic review on algorithms supports this recommendation by demonstrating the effectiveness of incorporating measurement instruments into algorithms to aid in decision-making of treatment and care by HCPs and standardizing practice (60).Additionally, this same review reports the common determinants and implementation strategies (60), that quality improvement teams can use to facilitate implementation of CPG recommendations.

Furthermore, the results have several applications to enhance care and outcomes. Firstly, as mentioned, they can guide HCPs in decision making through the implementation of the summary recommendations. Secondly, they can help organizationally, with auditing current practices, and subsequently could be used to develop opportunities for staff education and learning. Lastly, they can serve as a foundation for the development of a more comprehensive CPG. While developing *de novo* CPGs is time consuming, taking on average 2–3 years (63), to expedite the development process, the included high- and medium-quality CPGs can be adapted.

Related to methodological approaches to the adaptation of CPGs, the most commonly used are the GRADE-ADOLOPMENT (64) or ADAPTE (65), but 19 other adapt/adopt approaches have been identified (66). Adapting existing CPGs allows for contextualization (64), reduces time and resource requirements. The GRADE-ADOLOPMENT process has resulted in high-quality CPGs in non-pediatric reviews (67). However, none of the three adapted CPGs in our review employed a standardized approach to adaptation. This highlights the importance of using one of the methodological approaches to ensure the quality and trustworthiness of CPGs. Developers of CPGs must also go a step further and use available reporting checklists to ensure accurate reporting of the development process, which could include the AGREE II for *de novo* CPGs (27), or the RIGHT statement for adapted CPGs (68). In our review, only a small proportion of CPG development groups chose to use the AGREE II, either to guide development or reporting, indicating a need for more rigorous commitment to transparency and methodological rigor. A criticism of CPGs is taking too long to update (69), and this was supported by our review, however there are no clear guidelines of timing for updating (70). Reasons for this, may include lack of resources and funding. Similarly, and the proliferation of CPGs overtime in societies whose main business has become CPG development, can result in numerous CPGs that require updating simultaneously and can impact human and financial resource availability.

While HCPs rely on CPGs for combining evidence to make management decisions, quality and trustworthiness are often implicitly assumed. However, this review identified concerns regarding methodological quality of the CPG development process and the consistency of recommendations and supporting evidence. Using some of the IOM trustworthiness criteria (12), the following paragraphs will address these concerns. Despite these limitations, CPGs have an important role in consolidating the medical literature and provide new insights into patient care, which can ultimately improve patient outcomes in the PICU.

It is important to address methodological quality in the development process of the included CPGs, with a lack of use of rigorous and transparent methods. According to the IOM criteria for trustworthy CPGs (12) as applied to each step of the GRADE development process (71), the first critical step is to consider outcomes and prioritize their importance (71). However, none of the included CPGs performed this step. Next a comprehensive systematic review is an essential component of trustworthy CPG development (71) and is the fourth IOM criteria (12). However, none of the included CPGs considered the COnsensus-based Standards for the selection of health Measurement Instruments (COSMIN) standards for evaluating the psychometric properties and clinical utility of measurement instruments (72). Incorporating the COSMIN standards and using the established search filter (73), could have enhanced the comprehensiveness and robustness of the CPG development process. Additionally, there are existing reviews on measurement instruments for pain and sedation, and withdrawal that were not included in any of the current CPGs (74, 75). This may be attributed to limitations in the search strategies employed, which could be overcome by using a health information specialist to assist with the development of the search strategy. The presence of bias in some search strategies was another issue. For example, in the SCCM CPG, only the FLACC and COMFORT-B scales were included as terms for measurement instruments, without a comprehensive search for other relevant measurement instruments (44). This bias limits the scope and potential inclusion of other validated measurement instruments that could contribute to more comprehensive pain and sedation assessment.

Additionally, many of the included CPGs lacked patient and family involvement in the CPG development process, which is a trend among CPG development groups noted in another review (76). Patient and family involvement in evidence-based practice is crucial, and several research groups have emphasized the need for their inclusion to ensure their values and preferences are considered and understood as part of recommendation development (71, 77, 78). Additionally, the lack of importance placed on outcome generation across all CPGs, coupled with minimal patient involvement in the development process, is problematic. In our review, most CPG development groups did not include patient and/or parent representatives during the external review process and missed the opportunity to gain a broader and important perspective which the development group alone does not possess (79). To address this gap, future CPGs development groups should prioritize the inclusion of patients and families at various stages of development. The recently validated PANELVIEW tool provides a means for patients to assess their level of involvement in the CPG development process and CPG development groups should consider its use (80).

While our systematic review showed a convergence of recommendation across multiple CPGs, it also revealed a lack of consistency in the levels of SoR and CoE across the CPGs. Only 47% of CPGs provided information on the system used to determine the SoR and CoE. The overall consistency remains inadequate as demonstrated by other recent reviews (67, 81). This lack of consistency is concerning as it raises questions about the reliability and validity of the recommendations within CPGs. Moreover, many of the recommendations were based on minimal evidence, and the available evidence often did not encompass the population for which the CPGs were intended. For example, some recommendations based on evidence from adult populations or pediatric patients with specific conditions (e.g., cardiac surgery), may not apply to all children for whom the CPG was developed. This lack of generalizability compromises the applicability of the recommendations and highlights the need for more robust evidence that is representative of the target populations. Additionally, other CPGs were often used as supporting evidence for recommendations, however, this purports that these CPGs are well developed and evaluated the evidence in a rigorous manner which our review and others have shown is not always the case (14–16).

This review found a lack of description regarding the methods used to develop recommendations and how they translated these into SoRs. The review also highlighted many inconsistent and inconclusive recommendations across the included CPGs. This supports the need for more high-quality studies to increase the level of recommendation from that of expert opinion, conditional or very low. Lack of transparency is a challenge for understanding the rationale behind recommendations and makes it difficult to assess their quality. None of the CPGs, had openly available evidence to decisions tables. It is importance to enhance the availability of the evidence, so others can appraise the evidence for themselves. This transparency will contribute to reliable and credibly recommendations.

The challenges identified with the quality of development and the credibility of evidence, together highlight the need to formally appraise study quality in the CPG development process and the need for using standardized rating processes such as GRADE to produce solid recommendations. As mentioned, the next step should be the development and adaptation of a rigorous CPG on the management of pain, sedation, delirium, and IWS. This CPG should address the gaps in methodological quality of the previously developed CPGs and should take into consideration the gaps in the literature identified (44, 82).

## 5. Strengths and limitations

This is the first systematic review to assess quality and to synthesize CPG recommendations for pain, sedation, delirium, and IWS assessment and management. This systematic review has several strengths, firstly, comprehensive methods were employed to locate and assess CPGs related to the four conditions and their assessment and management, ensuring coverage of relevant guidance documents. Secondly, this systematic review used rigorous methods to assess the quality of the included CPGs, their recommendations, and the supporting evidence. The use of the AGREE II instrument allowed for an evaluation of the development process of CPGs, while the AGREE-REX was added to provide an extensive appraisal of recommendations. Furthermore, an assessment of the supporting evidence of each recommendation was undertaken. This rigorous approach to quality assessment allowed for the interpretation of trustworthiness of included CPGs. Overall, these strengths make the systematic review valuable for HCPs by providing them with summary recommendations.

There are certain limitations that should be acknowledged, the first, is that the IOM criteria were not utilized as part of our analysis, as has been done in other systematic reviews of CPGs (67, 83). However, the IOM criteria were considered and used to scaffold the discussion of trustworthiness.

Another limitation is the difficulty of accessing CPGs, as they are not always published or readily indexed in databases (20). To limit the potential accessibility and retrievability bias, the review used an exhaustive search strategy conducted by an expert librarian, with no language restrictions. This rigorous approach enhances the confidence in the review's comprehensiveness in capturing the available CPGs at the time of performing the searches.

The last limitation is related to the inclusion of other guidance document types which may have reduced quality due to lack of reporting or use of appropriate methodological methods. However, given the limited availability of CPGs at the time our systematic review was initiated, the inclusion of other guidance documents was deemed necessary. Our review highlights the need for more consistent terminology to differentiate among the various types of guidance documents (84) and improvements in methodological rigor (85). It is crucial to conduct a thorough assessment using appropriate tools, such as the AGREE II or other available tools (66, 86), before relying on any type of guidance document or CPG.

## 6. Conclusion

This systematic review evaluated 18 CPGs for the management of pain, sedation, delirium, and IWS in the PICU. Most CPGs and recommendations were medium-quality, as appraised by the AGREE II and AGREE-REX instruments. From six CPGs, a total of 170 recommendations were synthesized into 30 summary recommendations for the management of these four conditions to enhance our understanding of the quality and trustworthiness of these CPGs. The review identified large variations in the SoR and CoE across the synthesized summary recommendations. These are focused on medium-and high-quality CPGs and offer a concise minimum standard that PICUs teams can apply, allowing quality improvement teams to focus on long-term planning that larger-scale changes require. Utilizing implementation strategies and algorithm/protocolized care can facilitate the adoption of our summary recommendations. The applicability domain of the AGREE II instrument was particularly low emphasizing the importance of including practical implementation resources in CPGs to bridge the evidence-to-practice gap. The lack of involvement of patient and family in the development process is a notable shortcoming and future CPG development teams should prioritize their inclusion to capture their lived experiences, values and preferences. Addressing these two shortcomings will enhance the relevance and trustworthiness of the recommendations for clinical practice in the PICU. Robust and transparent methods should be employed during guideline development to enhance the credibility and usefulness of CPGs. Future research should focus on updating CPGs in a timely manner and ensuring HCPs have access to the latest high-quality CPGs and recommendations to provide optimal patient care in the PICU.

## Data Availability

The original contributions presented in the study are included in the article/**Supplementary Material**, further inquiries can be directed to the corresponding authors.
