# Supplementary material for: A systematic review of clinical practice guidelines and recommendations for the management of pain, sedation, delirium and iatrogenic withdrawal syndrome in pediatric intensive care
Source: Front Pediatr. 2023 Oct 6;11:1264717. doi: 10.3389/fped.2023.1264717 (PMC10587441; doi:10.3389/fped.2023.1264717)
Supplement: Supplementary file 1 [file Datasheet1.pdf]

Supplementary Material

A systematic review of clinical practice guidelines and recommendations for the management of pain, sedation, delirium and iatrogenic withdrawal syndrome in pediatric intensive care

Ibo MacDonald, Silvia Alvarado, Mark T. Marston, Luz Gomez Tovar, Vivianne Chanez, Eva Favre, Ying Gu, Alexia Trombert, Maria-Helena Perez, Anne-Sylvie Ramelet*

*** Correspondence:** Corresponding Author: anne-sylvie.ramelet@chuv.ch

Table S1: PICAR statement: inclusion and exclusion criteria 1
Table S2: Search strategy - Electronic databases 3
Table S3: Search strategy - guideline repositories 5
Table S4: Search strategy - professional societies/organizations 9
Table S5: Categories and sub-categories for recommendations 10
Table S6: Excluded references and reasons 11
Table S7: AGREE II raw scores by reviewers 18
Table S8: Recommendations and decision for inclusion or exclusion 19
Table S9: Synthesized pain recommendations – consistency across CPGs for SoR,and CoE, and review of evidence relevance and support 36

Table S1: PICAR statement: inclusion and exclusion criteria

Population, clinical indication(s), and condition(s)	**Study population**:<br>- Include: Children (newborn (>38 weeks gestations) to 18 years of age)<br>- Exclude: premature infants and adults<br><br>**Clinical indications**:<br>- Include: management of either pain (including postoperative, persistent and prolonged pain), sedation, delirium or IWS<br>- Exclude: Management specific to medications, chronic and procedural pain of short duration, procedural sedation provided in other care settings (e.g. dentistry, radiology, endoscopy) or for short duration<br><br>**Conditions**:<br>- Include: children in intensive care
Interventions	Any intervention focusing on the on-going management of either pain, sedation, delirium or IWS
Comparator(s), comparison(s), and (key) content	**Comparator/comparison:** Any<br>**Key content**:

	- If a broad population is included, the CPG must have separate recommendations for children<br>- The CPG can be implemented in the intensive care setting but does not need to be specifically developed for intensive care<br>- The CPG must include recommendations on assessment for either pain, sedation, delirium or IWS
**A**ttributes of eligible CPGs	**Language:** No restrictions<br>**Year of publication**: 2010 onward<br>**Setting**:<br>- Include: Applicable to pediatric intensive care, can be broad/general<br>- Exclude: CPGs developed specifically for other settings: neonatal intensive care units, emergency department, pre-operative/operating room<br><br>**Developing/publishing organization:**<br>- Include: CPGs issued or endorsed by international, national or regional societies/professional organizations, or governments from developed countries<br>- Exclude: CPGs that were developed by an individual organization (e.g. hospital) or unit within an organization<br>**Version**: Latest/newest version (preceding versions will be excluded)<br>**Type**: All guidance documents, including CPGs, consensus statements, practice/position recommendations/alerts/statements<br>**Quality score**: The AGREE II will be used to assess quality but will not be used as a criterion to determine eligibility for inclusion in this systematic review
**R**ecommendations characteristics and “other” considerations	**Recommendations:** CPGs must have at least one specific recommendation for assessment for either pain, sedation, delirium or IWS (either explicitly highlighted as a recommendation (primary) or noted in the body of the text (secondary - not explicitly identified as a recommendation))

Table S2: Search strategy - Electronic databases

The terms used to limit the search to guidelines are based on the search hedge Guidelines/Recommendations (revised 12/3/2015, consulted in August 2021) of the University of Texas School of Public Health (https://libguides.sph.uth.tmc.edu/search_filters/ovid_medline_filters), adapted by the health information specialist consulted (AT) and colleagues.

**Database and search strategy**	**Number of results**
**Embase.com**<br><br>('pain'/de OR 'postoperative pain'/de OR 'analgesia'/exp OR 'conscious sedation'/de OR 'hypnotic sedative agent'/de OR 'delirium'/de OR 'delirium assessment'/exp OR 'hyperactive delirium'/de OR 'hypoactive delirium'/de OR 'withdrawal syndrome'/de OR (pain OR discomfort OR analgesia OR sedation OR sedative* OR sedate* OR delirium OR delirious* OR withdrawal*):ti,kw) AND ('practice guideline'/de OR 'consensus development'/de OR ((expert* NEAR/3 opinion*) OR guideline* OR cpg* OR guidance OR ((position OR policy) NEAR/3 (paper* OR development)) OR ((practice OR clinical) NEAR/3 development) OR (practice NEAR/2 guide$) OR recommendation* OR consensus OR standards OR statement*):ti,kw) NOT ('adult'/exp NOT 'juvenile'/exp) AND [2010-3000]/py NOT ('conference abstract'/it OR 'conference review'/it) NOT ([animals]/lim NOT [humans]/lim)	11509<br>(26 May 2023)
**Medline ALL (Ovid)**<br>*Ovid MEDLINE(R) ALL 1946 to May 25, 2023*<br><br>(Pain/ OR Pain, Postoperative/ OR exp Analgesia/ OR Pain Management/ OR Conscious Sedation/ OR "Hypnotics and Sedatives"/ OR exp Confusion/ OR Substance Withdrawal Syndrome/ OR (pain OR discomfort OR analgesia OR sedation OR sedative* OR sedate* OR delirium OR delirious* OR withdrawal*).ti,kf.) AND ("Guidelines as Topic"/ OR "Practice Guidelines as Topic"/ OR exp Guideline/ OR Consensus/ OR ((expert* ADJ3 opinion*) OR guideline* OR cpg* OR guidance OR ((position OR policy) ADJ3 (paper* OR development)) OR ((practice OR clinical) ADJ3 development) OR (practice ADJ2 guide?) OR recommendation* OR consensus OR standards O(1)R statement*).ti,kf.) NOT (exp Adult/ NOT (exp Child/ OR exp Infant/ OR exp Adolescent/)) NOT (exp Animals/ NOT Humans/)	4962<br>(26 May 2023)

+ date limit 2010-current	
**CINAHL with Full Text EBSCO**<br><br>((MH "Pain") OR (MH "Postoperative Pain") OR (MH "Analgesia+") OR (MH "Pain Management") OR (MH "Conscious Sedation") OR (MH "Hypnotics and Sedatives") OR (MH "Confusion+") OR (MH "Substance Withdrawal Syndrome") OR TI(pain OR discomfort OR analgesia OR sedation OR sedative* OR sedate* OR delirium OR delirious* OR withdrawal*)) AND ((MH "Practice Guidelines") OR (MH "Consensus") OR TI((expert* N2 opinion*) OR guideline* OR cpg* OR guidance OR ((position OR policy) N2 (paper* OR development)) OR ((practice OR clinical) N2 development) OR (practice N1 guide#) OR recommendation* OR consensus OR standards OR statement*)) NOT ((MH "Adult+") NOT ((MH "Child+") OR (MH "Adolescence+"))) AND PY 2010-3000	3723<br>(26 May 2023)
**JBI EBP Database (Ovid)**<br><br>(sedation or sedative* or sedate* or pain or delirium or withdrawal).ti. AND (infant OR infants OR baby OR babies OR newborn* OR child* OR kid* OR juvenile OR preadolescen* OR adolescen* OR youth OR prepube* OR teenager* OR pediatric* OR paediatric* OR PICU).mp.<br><br>+ limit to<br>Best practice information sheets<br>Evidence summaries<br>Recommended practices<br><br>+ limit 2010-current	88<br>(26 May 2023)

Table S3: Search strategy - guideline repositories

Repository	Search strategy	Date
ECRI Guidelines Trust<br><br>https://guidelines.ecri.org/<br><br>*Note: at time of submission, ECRI Guidelines Trust is no longer accessible.*	pain OR analgesia OR sedation OR withdrawal OR delirium<br><br>Resource type: Guidance<br><br>139 hits	3 April 2022
Guideline Central<br><br>https://www.guidelinecentral.com/summaries/#summary-viewspecialty	Guidelines > Search panel<br><br>1. Search term: Pain + Specialty filter: Critical care = 23 hits<br>2. Search term: Pain + Specialty filter: Pediatrics = 92 hits<br>3. Analgesia = 58 hits<br>4. Sedation = 80 hits<br>5. Withdrawal = 82 hits<br>6. Delirium = 26 hits	3 April 2022
Guidelines International Network (G-I-N)<br>https://guidelines.ebmportal.com/	Search International Guidelines Library<br><br>1. Pain = 97 hits<br>2. Analgesia = 8 hits<br>3. Sedation = 9 hits<br>4. Withdrawal = 2 hits<br>5. Delirium = 17 hits	3 April 2022

TRIP<br>https://www.tripdatabase.com/	(title:pain OR title:analgesia OR title:sedation OR title:withdrawal OR title:delirium) AND (pediatric OR paediatric OR infant OR baby OR child OR kid OR adolescent OR teenager OR juvenile)<br><br>+ filter results: Guidelines = 239 hits	3 April 2022
Uptodate Society guideline links<br>http://www.uptodate.com/contents/search	1. Pain<br>• Topics reviewed for society guideline links: Evaluation and management of pain in children, Approach to the management of acute perioperative pain in infants and children, Pain control in the critically ill adult patient, Prevention and treatment of neonatal pain<br>• Society guideline links found: Society guideline links: Acute pain management<br><br>2. Sedation<br>• Topics reviewed for society guideline links: Selection of medications for pediatric procedural sedation outside of the operating room, Sedative-analgesic medications in critically ill adults: Selection, initiation, maintenance, and withdrawal, Procedural sedation in children outside of the operating room, Preparation for pediatric procedural sedation outside of the operating room, Pharmacologic agents for pediatric procedural sedation outside of the operating room, Sedative-analgesic medications in critically ill adults: Properties, dosage regimens, and adverse effects<br>• Society guideline links found:<br>Society guideline links: Procedural sedation in children<br>Society guideline links: Procedural sedation in adults<br>Society guideline links: Nonprocedural sedation<br><br>3. Delirium<br>• Topics reviewed for society guideline links: Emergence delirium and agitation in children, Diagnosis of delirium and confusional states, Delirium and acute confusional states: Prevention, treatment, and prognosis, Emergence delirium and agitation in children	3 April 2022

	• Society guideline links found: Society guideline links: Delirium and confusional states in older adults<br><br>4. Withdrawal<br>• Topics reviewed for society guideline links: Deprescribing, Opioid withdrawal in adults: Clinical manifestations, course, assessment, and diagnosis, Opioid withdrawal in adolescents, Sedative-analgesic medications in critically ill adults: Selection, initiation, maintenance, and withdrawal<br>• Society guideline links found: Society guideline links: Opioid use disorder and withdrawal	
CISMeF Bonnes pratiques<br><br>https://doccismef.chu-rouen.fr/dc/#env=bp	Simple search panel<br><br>((douleur.ti) OU (douleur.mc) OU (sédation.ti) OU (sédation.mc) OU (sevrage.ti) OU (sevrage.mc) OU (delirium.ti) OU (delirium.mc))<br><br>258 hits	3 April 2022
Infobanque des Guides de Pratique Clinique (GPC)<br><br>https://jouleamc.ca/cpg/homepage	1. Douleur = 58 hits<br>2. Pain = 89 hits<br>3. Analgésie = 12 hits<br>4. Analgesia = 15 hits<br>5. Sédation = 9 hits<br>6. Sedation = 9 hits<br>7. Delirium = 15 hits<br>8. Délir* = 15 hits<br>9. Sevrage = 28 hits<br>10. Withdrawal = 16 hits	20 December 2021
BIGG International database of GRADE guidelines<br>https://sites.bvsalud.org/bigg/en/biblio/	pain OR analgesia OR sedation OR withdrawal OR delirium<br>125 hits	June 3, 2023

NICE Evidence Search<br>https://www.evidence.nhs.uk/<br><br>*Note: at time of submission, NICE Evidence Search is no longer accessible.*	(pain or analgesia or sedation or withdrawal or delirium) and (pediatric or paediatric or infant or baby or child or kid or adolescent or teenager or juvenile)<br><br>Date from 01.01.2010 to 21.12.2021<br><br>Evidence type: guidance<br><br>2295 hits	21 December 2021

Table S4: Search strategy - professional societies/organizations

Organization website	Country/ Region	Search strategy	Date
American Association of Critical Care Nurses (AACN) https://www.aacn.org/	USA	Scrolled all the practice alerts https://www.aacn.org/clinical-resources/view-all-issues?category=practice-alerts	July 8 2022
European Society of Intensive Care Medicine (ESICM) https://www.esicm.org/	Europe	Resources -> Guidelines and consensus statements (reviewed them all) https://www.esicm.org/resources/guidelines-consensus-statements/	July 8 2022
European Society of Paediatric Neonatal Intensive Care (ESPNIC) https://www.espnic.eu/	Europe	Science -> Our publications -> My NCBI Collection – ESPNIC publications https://tinyurl.com/ESPNICpublications Filtered since 2010 = 46 hits (reviewed them all)	June 12 2023
National Institute for Health and Care Excellence (NICE) www.nice.org.uk	UK	Guidance -> View Guidance -> View all Guidance -> Published https://www.nice.org.uk/guidance/published Type: Guidance, NICE advice, Quality standard Last updated date: from date 01.01.2010 Filter by title or keyword search panel: 1. Pain = 28 hits 2. Sedation = 2 hits 3. Delirium = 2 hits 4. Withdrawal = 0 hits	July 8 2022
Registered Nurses' Association of Ontario (RNAO) https://rnao.ca/	Canada	BPG & BPSO -> Best practice guidelines -> Filter: clinical https://rnao.ca/bpg/guidelines Reviewed all 19 « clinical » practice guidelines	June 12 2023
Royal College of Nursing https://www.rcn.org.uk/	UK	Website search box 1. pain = 61 hits 2. sedation = 5 hits 3. delirium = 6 hits 4. withdrawal = 27 hits	July 16, 2023
Scottish Intercollegiate Guidelines Network (SIGN) https://www.sign.ac.uk/	Scotland	Our Guidelines https://www.sign.ac.uk/our-guidelines/ Looked at all under "Current"	July 8 2022
Société de Réanimation de Langue Française (SRLF) https://www.srlf.org/	France	Recommandations, Référentiels et EPP (searched all)	July 8 2022

		https://www.srlf.org/recommandations-referentiels-epp	
Société Française d'Anesthésie et de Réanimation (SFAR) https://sfar.org	France	Recommandations https://sfar.org/recommandations/ Chronologie: browsed through all years from 2010	June 18 2023
Société Suisse de Pédiatrie https://www.paediatrieschweiz.ch/fr/	Switzerland	Search all « documents ». Documents -> Browsed through all https://www.paediatrieschweiz.ch/fr/documents/	July 8 2022
Société Suisse de Médecine Intensive (SSMI) https://www.sgi-ssmi.ch/fr	Switzerland	Douleur: 9 hits<br>Sédation: 14 hits<br>Delirium: 0 hits<br>Délire: 11 hits<br>Sevrage: 0 hits	16 July 2023
Society of Critical Care Medicine (SCCM) https://www.sccm.org/Home	USA	Clinical resources -> Guidelines -> Access All SCCM Guidelines -> Browsed through all https://www.sccm.org/Clinical-Resources/Guidelines/Guidelines	July 8 2022
World Federation of Pediatric Intensive and Critical Care Societies (WFPICCS) https://wfpiccs.org	Worldwide	Resources -> Health professionals https://wfpiccs.org/health-professionals/	July 8 2022

Table S5: Categories and sub-categories for recommendations

**Protocol**	**Revised**
1. Prevention	
2. Assessment	1. Assessment:<br>a) Assessment/screening by:<br>i. HCPs<br>ii. families<br>b) Documentation<br>c) Diagnosis
3. Management<br>a) Pharmacological<br>b) Non-pharmacological	2. Management/intervention:<br>a) Prevention<br>b) Pharmacological<br>c) Non-pharmacological<br>d) Follow-up/discharge<br>e) Planning
	3. Implementation
	4. Education
	5. Organizational/policy

Table S6: Excluded references and reasons

Reference	Reason for Exclusion
AACN cracks down on delirium. Nursing. 2012;42(5):24-.	1
Aldecoa C, Bettelli G, Bilotta F, Sanders RD, Audisio R, Borozina A. European Society of Anaesthesiology evidence-based and consensus-based guideline on postoperative delirium. 2017;34(4) :192-214.	2
Acute pain guidelines. South African Family Practice. 2016;58(5):20-41.	4
Acute Pain Guidelines. Professional Nursing Today. 2017;21(2):29-46.	6
Agbeko RS, Argent A, MacLaren G. Mind over matter? Pain, withdrawal and sedation in paediatric critical care. Intensive Care Medicine. 2016;42(8):1261-3	1
Alcántara Montero A, González Curado A. New American Pain Society Guideline on postoperative pain. Revista de la Sociedad Espanola del Dolor. 2016;23(3):164-5.	1
Alford DP, Krebs EE, Chen IA, Nicolaidis C, Bair MJ, Liebschutz J. Update in pain medicine. Journal of General Internal Medicine. 2010;25(11):1222-6.	1
American Pain Society publishes guideline for post-surgical pain management. Same-Day Surgery. 2016;40(4):44-5.	1
American Society for Pain Management N, Emergency Nurses A, American College of Emergency P, American Pain S. Optimizing the treatment of pain in patients with acute presentations. Policy statement. Ann Emerg Med. 2010;56(1):77-9.	1
American Society of Anesthesiologists Task Force on Acute Pain M. Practice guidelines for acute pain management in the perioperative setting: an updated report by the American Society of Anesthesiologists Task Force on Acute Pain Management. Anesthesiology. 2012;116(2):248-73.	2
Ancora G, Lago P, Garetti E, Merazzi D, Savant Levet P, Bellieni CV, et al. Evidence-based clinical guidelines on analgesia and sedation in newborn infants undergoing assisted ventilation and endotracheal intubation. Acta Paediatrica. 2019;108(2):208-17.	2
Anonymous. Statement of withdrawal. J Occup Environ Hyg. 2017;14(2):D28.	1
Anonymous. Article Withdrawal Statement. Int J Neurosci. 2016;126(8):i-viii.	1
Anonymous. RCN issues guidance on pain assessment and management. Nurs Stand. 2015;30(2):10	1
Anonymous. Guideline Quick View: Moderate Sedation. AORN Journal. 2021;114(2):203-8.	1
Anonymous. Assessment and Management of Delirium Across the Life Span. Critical Care Nurse. 2017;37(2):112.	1
Aorn. Guideline at a Glance: Moderate Sedation/Analgesia. AORN Journal. 2017;105(6):638-42.	1
APS Releases Exhaustive Guidelines for Post-Op Pain. Operating Theatre Journal. 2016(306):8-.	1
Aubrun F, Nouette Gaulain K, Fletcher D, Belbachir A, Beloeil H, Carles M, et al. Revision of expert panel guidelines on postoperative pain management. Anesth Reanim. 2016;2(6):421-30.	4

Aubrun F, Nouette Gaulain K, Fletcher D, Belbachir A, Beloeil H, Carles M, et al. Revision of expert panel guidelines on postoperative pain management. Douleurs. 2017;18(1):34-43.	4
Azimaraghi O, Smith V, Sauer WJ, Alpert JE, Eikermann M. Agitated Patients in the Intensive Care Unit: Guidelines for Causal Rather Than Symptomatic Treatment are Warranted. J Intensive Care Med. 2023;38(2):238-40.	
Baker DW. Statement on Pain Management: Understanding How Joint Commission Standards Address Pain. Jt Comm Perspect. 2016;36(6):10-2.	1
Baulch I. Assessment and management of pain in the paediatric patient. Nurs Stand. 2010;25(10):35-40.	1
Bell L. Pain scales and pain management. American Journal of Critical Care. 2012;21(4):260.	1
Beloeil H, Bonnet F, Prospect working g. From universal postoperative pain recommendations to procedure-specific pain management. Anaesth Crit Care Pain Med. 2018;37(4):305-6.	1
Brand K. Pain assessment in children. Anaesthesia and Intensive Care Medicine. 2022;23(5):260-3.	1
Brasher C, Gafsous B, Dugue S, Thiollier A, Kinderf J, Nivoche Y, et al. Postoperative pain management in children and infants: An update. Pediatric Drugs. 2014;16(2):129-40.	1
Carbajal R, Gatterre P, Rambaud J, de Suremain N. [Pain and newborn screening]. Archives de Pediatrie. 2016;23(3):229-31.	1
Carnevale FA. Advancing analgesia and sedation for critically ill children. Pediatric Intensive Care Nursing. 2018;19(1):1-.	2
Coetzee JF. Sedation guidelines: A record of sedation scores is essential. Southern African Journal of Anaesthesia and Analgesia. 2010;16(4):10-1.	1
Cooney MF. Postoperative Pain Management: Clinical Practice Guidelines. J Perianesth Nurs. 2016;31(5):445-51	1
Coplin L. Pediatric Groups Update Sedation Clinical Guidelines. Same-Day Surgery. 2016;40(9):106-7.	1
Correll DJ, Golembiewski J, Pizzi LJ. Focus on safety: Managing postoperative pain. Clinical Advisor. 2018;21(4):35-42.	1
Cote CJ. American Academy of Pediatrics sedation guidelines: are we there yet? Arch Pediatr Adolesc Med. 2012;166(11):1067-9.	1
Croke L. Guideline for care of the patient receiving moderate sedation/analgesia. AORN Journal. 2021;113(6):P4-P6.	1
Czarnecki ML, Turner HN, Collins PM, Doellman D, Wrona S, Reynolds J. Procedural pain management: a position statement with clinical practice recommendations. Pain Management Nursing. 2011;12(2):95-111.	2
Davis D, Searle SD, Tsui A. The Scottish Intercollegiate Guidelines Network: risk reduction and management of delirium. Age Ageing. 2019;48(4):485-8.	1
Defontaine A, Ecoffey C. [Pain in children: sedation and analgesia]. Rev Prat. 2013;63(7):1019-23.	1
Drew D, Gordon D, Renner L, Morgan B, Swensen H, Manworren R, et al. The use of "as-needed" range orders for opioid analgesics in the management of pain: a consensus statement of the American Society of Pain Management Nurses and the American Pain Society. Pain Management Nursing. 2014;15(2):551-4.	2

Drew DJ, Gordon DB, Morgan B, Manworren RCB. "As-Needed" Range Orders for Opioid Analgesics in the Management of Pain: A Consensus Statement of the American Society for Pain Management Nursing and the American Pain Society. Pain Management Nursing. 2018;19(3):207-10.	2
Dries DJ. Sedation. Air Medical Journal. 2014;33(2):55-7.	1
Elmoheen A, Nazal AF, Zubaidi O, Siddiqui UA, Alhatou M. Expert review and recommendations for the management of acute, chronic, and neuropathic pain in Qatar. Qatar Medical Journal. 2021;2021(2):19.	5
Etzel-Hardman D. Pediatric sedation and distraction. J Pediatr Nurs. 2011;26(2):172-3.	1
Fendrychová J. Adaptovaný klinický doporučený postup: zavádění a ošetřování periferních žilních vstupů u novorozenců a kojenců. Pediatrie pro praxi. 2020;21(3):206-9.	5
Fonsmark L, Hein L, Nibroe H, Bundgaard H, de Haas I, Iversen S, et al. Danish national sedation strategy. Targeted therapy of discomfort associated with critical illness. Danish Society of Intensive Care Medicine (DSIT) and the Danish Society of Anesthesiology and Intensive Care Medicine (DASAIM). Dan Med J. 2015;62(4):C5052.	3
Freys S, Erlenwein J, Koppert W, Meißner W, Pogatzki-Zahn E, Schwenk W, et al. Vereinbarung zur Organisation der Schmerztherapie chirurgischer Patienten des Berufsverbandes Deutscher Anästhesisten und des Berufsverbandes der Deutschen Chirurgen (Neufassung 2019). Der Chirurg. 2019;90(8).	1
Frost EAm. Upgrades for Procedural Sedation Standards. Topics in Pain Management. 2012;28(3):9-.	3
Gal DB, Clyde CO, Colvin EL, Colyer J, Ferris AM, Figueroa MI, et al. Management of routine postoperative pain for children undergoing cardiac surgery: a Paediatric Acute Care Cardiology Collaborative Clinical Practice Guideline. Cardiol Young. 2022;32(12):1881-93.	2
Galinkin J, Koh JL, Committee on D, Section On A, Pain M, American Academy of P. Recognition and management of iatrogenically induced opioid dependence and withdrawal in children. Pediatrics. 2014;133(1):152-5.	1
Gangopadhyay M. 3.5 Management of Delirium: Current Evidence and Recommendations. Journal of the American Academy of Child & Adolescent Psychiatry. 2018;57:S6-S.	1
Garcia JBS, Bonilla P, Kraychete DC, Flores FC, Valtolina EDPd, Guerrero C. Aprimorar o controle da dor no pós-operatório na América Latina. Revista Brasileira de Anestesiologia. 2017;67:395-403.	1
Garten L, Demirakca S, Harth I, Huth R, Kumpf M, Schindler M, et al. Analgesie, Sedierung und Delirmanagement–Die DAS-Leitlinie 2015: Kinder und Neugeborene. AINS-Anästhesiologie· Intensivmedizin· Notfallmedizin· Schmerztherapie. 2015;50(11/12):712-22.	1
Guideline Summary: Moderate Sedation/Analgesia. AORN Journal. 2016;103(5):512-6.	1
Herr K, Coyne PJ, Ely E, Gelinas C, Manworren RCB. ASPMN 2019 Position Statement: Pain Assessment in the Patient Unable to Self-Report. Pain Management Nursing. 2019;20(5):402-3.	1

Herr K, Coyne PJ, McCaffery M, Manworren R, Merkel S. Pain assessment in the patient unable to self-report: position statement with clinical practice recommendations. Pain Management Nursing. 2011;12(4):230-50.	4
Houweling PL, Molag ML, van Boekel RL, Verbrugge SJ, van Haelst IM, Hollmann MW. ['Postoperative pain treatment' practice guideline revised]. Ned Tijdschr Geneeskd. 2013;157(49):A7005.	1
Irwin SA, Pirrello RD, Hirst JM, Buckholz GT, Ferris FD. Clarifying delirium management: practical, evidenced-based, expert recommendations for clinical practice. Journal of Palliative Medicine. 2013;16(4):423-35.	1
Joint Commission revises pain management standard. Same-Day Surgery. 2015;39(2):23-.	1
Joint Commission Updates Pain Management Standards. Oriental Medicine. 2015:17-.	1
Joshi GP, Kehlet H, Group PW. Guidelines for perioperative pain management: need for re-evaluation. British Journal of Anaesthesia. 2017;119(4):703-6.	1
Kehlet H. Updated pain guidelines: what is new? Anesthesiology. 2012;117(6):1397-8; author reply 8.	1
Křikava I, Kubricht V, Lejčko J, Málek J, Ševčík P, Štourač P, et al. Recommended procedure – treatment of acute postoperative pain. Anesteziologie a Intenzivni Medicina. 2022;33(2):111-20.	1
Lago P, Allegro A, Heun N. Improving newborn pain management: systematic pain assessment and operators' compliance with potentially better practices. Journal of clinical nursing. 2014;23(3):596-9.	1
Leentjens AF, Molag ML, Van Munster BC, De Rooij SE, Luijendijk HJ, Vochteloo AJ, et al. Changing perspectives on delirium care: the new Dutch guideline on delirium. Journal of Psychosomatic Research. 2014;77(3):240-1.	1
Likar R, Jaksch W, Aigmuller T, Brunner M, Cohnert T, Dieber J, et al. [Interdisciplinary position paper "Perioperative pain management"]. Schmerz. 2017;31(5):463-82.	2
Luz C, Ensberg M, Zhou S. Delirium Assessment and Management. Critical Care Nurse. 2012;32(1):79-82.	3
Marin T. Delirium in children : Prevention and management in postoperative care. 2021.	2
Marsh J, Alexander E, Allen J. Update on the Prevention and Treatment of Intensive Care Unit Delirium. AACN Advanced Critical Care. 2021;32(1):5-10.	1
Martin J, Heymann A, Basell K, Baron R, Biniek R, Burkle H, et al. Evidence and consensus-based German guidelines for the management of analgesia, sedation and delirium in intensive care--short version. German Medical Science. 2010;8:Doc02.	4
McArthur L. Sedation for infants and children. Nurs Times. 2011;107(18):18.	1
McFeely JE. Assessment, Prevention, and Treatment of Delirium in the ICU. Critical Care Alert. 2015;23(2):9-11.	1
Misiolek H, Cettler M, Woron J, Wordliczek J, Dobrogowski J, Mayzner-Zawadzka E. The 2014 guidelines for post-operative pain management. Anestezjol. 2014;46(4):221-44.	1
Misiolek H, Zajaczkowska R, Daszkiewicz A, Woron J, Dobrogowski J, Wordliczek J, et al. Postoperative pain management - 2018 consensus statement of the Section of Regional Anaesthesia and Pain Therapy of the	2

Polish Society of Anaesthesiology and Intensive Therapy, the Polish Society of Regional Anaesthesia and Pain Therapy, the Polish Association for the Study of Pain and the National Consultant in Anaesthesiology and Intensive Therapy. Anestezjol. 2018;50(3):173-99.	
MNA position statement on conscious sedation. Massachusetts Nurse Advocate. 2010;81(8):6-7.	1
Mungiu OC. Pain management: strategic guidelines. Rev Med Chir Soc Med Nat Iasi. 2014;118(4):893-5.	1
News A. Aafp, Acp Release New Acute Pain Clinical Guideline. Ann Fam Med. 2020;18(6):565-6.	1
New Guidelines for Moderate Procedural Sedation and Analgesia. AACN Bold Voices. 2018:15-.	1
Ohio Guideline for the Management of Acute Pain Outside of Emergency Departments. Momentum (Ohio Board of Nursing). 2016;14(1):24-5.	5
O'Mahony R, Murthy L, Akunne A, Young J. Synopsis of the National Institute for Health and Clinical Excellence guideline for prevention of Delirium. Annals of Internal Medicine. 2011;154(11):746-51.	1
Pain, Agitation, Delirium Guidelines Expand Nurses' Toolkit. AACN Bold Voices. 2014;6(4):7-.	1
Pain management. Journal of Hospital Medicine. 2010;5:63-4.	1
Pasero C. Opioid-induced sedation and respiratory depression: evidence-based monitoring guidelines. J Perianesth Nurs. 2012;27(3):208-11.	1
Patino M, Samuels P, Mahmoud M. Pediatric sedation outside the operating room. Int Anesthesiol Clin. 2013;51(2):127-46.	1
Páramo-Cano T, Ortiz MI, Gómez-Busto FJ, Espinoza-Ramírez AL. Management of procedural pain in children. Current pediatric reviews. 2021.	1
Position Statement: 94-1 -- Role of the Registered Nurse in the Management of Patients Receiving Moderate Sedation, Anesthetic Agents or Neuromuscular Blocking (paralytic) Agents for Therapeutic or Diagnostic Procedures. ASBN Update. 2017;21(3):20-1.	1
Price C, Lee J, Taylor AM, Baranowski AP, British Pain S. Initial assessment and management of pain: a pathway for care developed by the British Pain Society. British Journal of Anaesthesia. 2014;112(5):816-23.	2
Putnam K. Guideline for care of the patient receiving moderate sedation. AORN Journal. 2015;102(5):P10-P2.	1
Rajapakse D. WHO Guidelines on Persisting Pain in Children. European Journal of Palliative Care. 2013;20(4):171-.	1
Revised guidelines for safe sedation of pediatric patients. Clinical Advisor. 2016;19(8):15-.	5
Revised Guidelines Identify Tools for Pain, Agitation, Delirium Assessment and Monitoring. AACN Bold Voices. 2013;5(5):12-.	1
Riker RR, Fraser GL. The New Practice Guidelines For Pain, Agitation, and Delirium. American Journal of Critical Care. 2013;22(2):153-7.	1
Rundshagen I, American College of Critical Care M. [Pain, agitation and delirium. Amended 2013 guidelines of the American College of Critical Care Medicine]. Anaesthesist. 2013;62(11):914-8.	1
Sabatowski R, Arnold B, Irnich D, Pfingsten M, Schiltenwolf M. Pain medicine: Clear guidelines. Deutsches Arzteblatt International. 2016;113(47):A2161-A3.	1

Salgado-Cadena Dr BV. Treatment guidelines in pediatric pain. Revista Mexicana de Anestesiologia. 2012;35:S172-S4.	1
Scholten W. Developing 'fully evidenced' paediatric pain guidelines. Eur J Hosp Pharm-S P. 2013;20(5):262-3.	1
Schug SA, Palmer GM, Scott DA, Halliwell R, Trinca J. Acute pain management: scientific evidence, 2015. Medical Journal of Australia. 2016;204(8):315-7.	4
Soiza RL, Myint PK. The Scottish Intercollegiate Guidelines Network (SIGN) 157: Guidelines on Risk Reduction and Management of Delirium. Medicina (Kaunas). 2019;55(8):15.	1
Steiner LA. Postoperative delirium guidelines: The greater the obstacle, the more glory in overcoming it. Eur J Anaesthesiol. 2017;34(4):189-91.	1
Subspecialty Group of Emergency Medicine TSoPCMA, Subspecialty Group of Pediatrics TSoEMhMA, Pediatric Professional Committee TSoCCMCMDA. [Experts' consensus on sedation and analgesia in pediatric intensive care unit(2013)]. Zhonghua Er Ke Za Zhi. 2014;52(3):189-93.	4
Surgery-Specific Pain Management Guidelines. AACN Bold Voices. 2018;10(12):19-.	1
Taiwan Society Of Anesthesiologists S. Clinical Guidance of Mild to Moderate Sedation-An Expert Consensus by Taiwan Society of Anesthesiologists. Asian J Anesthesiol. 2020;58(2):57-60.	5
Tang B, Wang XT, Chen WJ, Zhu SH, Chao YG, Zhu B, et al. [Experts consensus on the management of delirium in critically ill patients]. Chung Hua Nei Ko Tsa Chih. 2019;58(2):108-18.	3
Taskforce DAS, Baron R, Binder A, Biniek R, Braune S, Buerkle H, et al. Evidence and consensus based guideline for the management of delirium, analgesia, and sedation in intensive care medicine. Revision 2015 (DAS-Guideline 2015) - short version. Ger Med Sci. 2015;13:Doc19.	4
Teegarden BMT, Prough DS. Delirium: Getting back on track. Critical Care Medicine. 2016;44(7):1265-6.	1
Vittinghoff M, Lonnqvist PA, Mossetti V, Heschl S, Simic D, Colovic V, et al. Postoperative pain management in children: Guidance from the pain committee of the European Society for Paediatric Anaesthesiology (ESPA Pain Management Ladder Initiative). Paediatric Anaesthesia. 2018;28(6):493-506.	1
Walker SM. Pain after surgery in children: clinical recommendations. Curr Opin Anaesthesiol. 2015;28(5):570-6.	1
Wall AB. Pain Management Guidelines. AACN Bold Voices. 2015;7(1):21-.	1
Williams K. Guidelines in Practice: Moderate Sedation and Analgesia. AORN J. 2022;115(6):553-64	2
Wolf A, Müller A, Weiß B, Spies C. S3 guideline: Management of delirium, analgesia and sedation in the intensive care. Anasthesiologie und Intensivmedizin. 2016;57(1):41-4.	1
Wong IMJ, Thangavelautham S, Loh SCH, Ng SY, Murfin B, Shehabi Y. Sedation and Delirium in the Intensive Care Unit-A Practice-Based Approach. Ann Acad Med Singapore. 2020;49(4):215-25.	1
Wrona SK, Quinlan-Colwell A, Brown L, Enricoso Jannuzzi RG. Procedural Pain Management: Clinical Practice Recommendations. Pain Manag Nurs. 2022;23(5):583-4.	2

Xu F, Qian SY. [Interpretation of experts' consensus on sedation and analgesia for children in pediatric intensive care unit of China (2018)]. Zhonghua Er Ke Za Zhi. 2019;57(5):336-7.	1
Yerra S, Yarra P. Best practices to prevent delirium in hospitalised patients. Evid Based Nurs. 2020;26:26.	1
Young J, Murthy L, Westby M, Akunne A, O'Mahony R, Guideline Development G. Diagnosis, prevention, and management of delirium: summary of NICE guidance. BMJ. 2010;341:c3704.	1
Yuhico AM, Collins J. Managing paediatric pain in general practice. Medicine Today. 2014;15(10):26-32.	5
1 = wrong publication type<br>2 = wrong outcomes<br>3 = wrong population<br>4 = older version of CPG<br>5 = wrong setting<br>6 = Full-text not available	

Table S7: AGREE II raw scores by reviewers

	SARNePI (2022)			AWMF			RNAO			NVA			ANZCA			NvVP			APS & ASA			PANDEM			ÖGARI		
**Reviewer (R)**	R1	R2	R3	R1	R2	R3	R1	R2	R3	R1	R2	R3	R1	R2	R3	R1	R2	R3	R1	R2	R3	R1	R2	R3	R1	R2	R3
**Domain 1: Scope and purpose**																											
1. The overall objective(s) of the guideline is (are) specifically described	6	3	6	7	7	7	4	6	7	6	6	7	5	4	6	6	6	7	7	4	7	6	7	6	5	7	6
2. The health question(s) covered by the guideline is (are) specifically described	7	7	6	7	4	7	5	4	7	6	5	4	2	1	2	5	3	6	5	2	7	5	6	5	5	5	5
3. The population (patients, public, etc.) to whom the guideline is meant to apply is specifically described	6	7	6	6	5	7	6	4	6	4	3	7	2	1	2	7	2	6	6	2	7	6	7	5	7	7	7
**Domain 2: Stakeholder involvement**																											
4. The guideline development group includes individuals from all relevant professional groups	6	7	7	5	3	5	5	5	6	5	4	7	5	6	4	5	5	7	7	6	7	5	6	5	5	5	5
5. The views and preferences of the target population (patients, public, etc.) have been sought	3	7	1	4	1	1	1	3	1	4	3	6	1	1	1	3	4	6	1	1	1	1	1	1	1	1	3
6. The target users of the guideline are clearly defined	6	7	5	2	2	3	7	7	7	6	2	1	4	2	6	6	7	7	5	4	7	2	1	2	6	6	6
**Domain 3: Rigor of development**																											
7. Systematic methods were used to search for evidence	6	7	5	5	6	7	6	7	7	7	7	7	4	6	3	4	4	7	2	2	4	5	6	3	3	6	4
8. The criteria for selecting the evidence are clearly described	6	7	5	5	6	7	6	4	7	3	3	1	3	2	3	4	4	5	2	1	5	2	5	2	3	6	4
9. The strengths and limitations of the body of evidence are clearly described	6	7	6	5	5	5	3	5	6	6	6	7	3	4	3	3	2	7	4	1	2	4	3	4	2	3	3
10. The methods for formulating the recommendations are clearly described	5	7	7	7	7	7	3	6	6	2	2	1	2	1	3	3	1	6	6	7	7	6	5	5	4	5	3
11. The health benefits, side effects, and risks have been considered in formulating the recommendations	5	7	6	5	4	4	3	6	5	5	7	1	4	5	5	3	1	6	3	5	6	4	5	5	3	3	3
12. There is an explicit link between the recommendations and the supporting evidence	5	7	6	7	7	4	5	7	7	6	7	5	4	4	6	5	3	6	7	4	6	7	7	7	5	2	6
13. The guideline has been externally reviewed by experts prior to its publication	5	7	4	4	1	6	2	1	5	3	2	1	6	4	5	4	3	5	3	3	7	1	1	1	4	6	1
14. A procedure for updating the guideline is provided	6	7	6	7	6	6	6	7	7	6	7	4	6	7	6	6	2	5	6	5	1	2	5	2	6	7	6
**Domain 4: Clarity of presentation**																											
15. The recommendations are specific and unambiguous	6	7	6	6	6	5	6	7	7	6	6	3	5	6	6	6	2	6	5	4	4	6	7	5	5	4	6
16. The different options for management of the condition or health issue are clearly presented	6	7	6	7	7	4	7	5	7	5	6	3	5	6	6	6	1	6	4	2	4	7	7	7	5	1	6
17. Key recommendations are easily identifiable	7	7	6	6	6	7	7	7	7	7	7	7	6	6	7	7	4	7	5	7	5	7	7	7	5	6	6
**Domain 5: Applicability**																											
18. The guideline describes facilitators and barriers to its application	2	5	2	2	1	3	2	5	6	3	4	1	3	1	4	2	1	3	1	1	1	1	5	1	1	1	2
19. The guideline provides advice and/or tools on how the recommendations can be put into practice	2	4	2	6	6	7	6	7	7	4	5	1	2	2	2	3	5	5	1	1	1	1	1	1	4	1	3
20. The potential resource implications of applying the recommendations have been considered	1	3	1	2	1	3	2	6	4	2	4	5	2	1	2	1	1	1	1	1	1	1	1	1	2	1	1
21. The guideline presents monitoring and/or auditing criteria	3	1	1	1	3	5	6	7	7	4	2	1	1	1	1	4	1	1	1	1	1	1	6	1	3	1	4
**Domain 6: Editorial independence**																											
22. The views of the funding body have not influenced the content of the guideline	1	1	1	7	6	7	2	1	6	2	7	4	4	7	5	2	4	4	3	7	7	1	6	1	1	2	2
23. Competing interests of guideline development group members have been recorded and addressed	6	7	6	6	7	7	4	5	6	5	6	5	4	7	5	4	2	2	7	7	7	3	6	3	6	1	5
**Overall guideline assessment**																											
1. Rate the overall quality of this guideline	5	6	4	6	4	5	5	7	6	5	5	5	4	3	5	5	3	6	4	3	6	5	5	4	3	3	4
2. I would recommend this guideline for use	Y	Y	YM	Y	Y	Y	YM	Y	Y	YM	MY	YM	YM	YM	YM	YM	N	Y	N	N	YM	YM	Y	YM	N	N	YM

	ESPNIC			APA			SFAR			CMA, CSMS & SCCP			SSPM			SARNePI (2014)			ASPMN			AACN			PSAIT		
**Reviewer (R)**	R1	R2	R3	R1	R2	R3	R1	R2	R3	R1	R2	R3	R1	R2	R3	R1	R2	R3	R1	R2	R3	R1	R2	R3	R1	R2	R3
**Domain 1: Scope and purpose**																											
1. The overall objective(s) of the guideline is (are) specifically described	7	7	7	6	7	5	2	2	7	6	6	6	7	6	7	4	7	7	7	7	7	4	5	1	2	1	1
2. The health question(s) covered by the guideline is (are) specifically described	3	3	6	6	5	5	7	7	2	5	3	5	7	6	2	3	5	7	1	2	7	1	1	1	3	1	1
3. The population (patients, public, etc.) to whom the guideline is meant to apply is specifically described	6	3	6	7	7	6	4	5	1	6	4	5	5	2	4	5	6	7	6	5	6	3	2	1	2	2	1
**Domain 2: Stakeholder involvement**																											
4. The guideline development group includes individuals from all relevant professional groups	2	7	5	4	6	4	3	5	1	5	2	2	4	4	2	4	5	6	1	2	5	1	1	1	2	2	1
5. The views and preferences of the target population (patients, public, etc.) have been sought	2	2	1	1	7	4	3	7	1	1	1	1	1	1	1	1	1	1	1	1	1	1	1	1	1	1	1
6. The target users of the guideline are clearly defined	7	7	4	6	6	5	2	1	1	5	1	3	4	7	7	2	1	1	2	2	6	2	2	1	4	2	1
**Domain 3: Rigor of development**																											
7. Systematic methods were used to search for evidence	6	6	7	3	6	3	2	1	1	4	2	3	2	4	2	3	3	3	1	1	1	1	1	1	2	2	1
8. The criteria for selecting the evidence are clearly described	4	2	6	3	3	3	1	1	1	3	2	1	1	2	2	1	1	1	1	1	1	1	1	1	1	1	1
9. The strengths and limitations of the body of evidence are clearly described	5	4	2	3	2	4	3	3	1	3	1	1	1	3	2	2	2	1	1	2	1	1	1	2	2	2	1
10. The methods for formulating the recommendations are clearly described	3	2	6	6	7	5	6	7	3	3	1	2	2	2	2	2	2	1	1	1	1	1	1	1	3	2	1
11. The health benefits, side effects, and risks have been considered in formulating the recommendations	3	3	6	5	3	3	7	7	3	5	4	2	2	5	2	4	3	3	1	4	2	2	2	1	2	2	5
12. There is an explicit link between the recommendations and the supporting evidence	5	5	5	6	4	6	5	3	1	5	4	3	1	3	2	5	1	2	6	6	2	5	4	2	3	2	2
13. The guideline has been externally reviewed by experts prior to its publication	5	5	1	1	1	3	7	7	1	1	1	1	1	4	1	1	1	1	1	1	1	1	1	1	1	1	1
14. A procedure for updating the guideline is provided	1	1	1	6	1	3	1	1	1	5	1	1	1	1	1	1	1	1	1	2	1	1	1	1	1	1	1
**Domain 4: Clarity of presentation**																											
15. The recommendations are specific and unambiguous	6	6	5	6	7	6	6	6	2	7	4	4	3	5	3	5	5	6	5	5	2	5	4	2	4	5	2
16. The different options for management of the condition or health issue are clearly presented	5	6	7	6	1	6	5	6	2	5	5	5	4	4	3	6	6	6	5	5	3	3	2	1	3	3	3
17. Key recommendations are easily identifiable	5	7	1	7	7	5	6	5	3	6	4	3	1	3	3	5	5	1	6	7	1	7	7	2	5	5	1
**Domain 5: Applicability**																											
18. The guideline describes facilitators and barriers to its application	1	2	3	4	1	4	1	1	1	4	1	1	2	2	3	1	1	5	1	1	2	2	1	1	1	1	1
19. The guideline provides advice and/or tools on how the recommendations can be put into practice	2	7	3	4	6	2	2	3	1	6	1	1	1	2	2	2	2	2	2	6	2	5	5	3	2	1	1
20. The potential resource implications of applying the recommendations have been considered	1	1	1	4	7	2	1	2	1	1	1	1	1	1	2	1	1	1	1	1	1	1	1	1	1	1	1
21. The guideline presents monitoring and/or auditing criteria	1	5	1	4	7	2	1	1	1	2	2	2	2	1	2	1	1	1	1	1	1	1	1	1	1	1	1
**Domain 6: Editorial independence**																											
22. The views of the funding body have not influenced the content of the guideline	7	7	7	5	5	1	7	7	1	4	1	5	7	7	2	2	1	7	1	1	1	1	1	5	4	6	7
23. Competing interests of guideline development group members have been recorded and addressed	3	5	6	5	7	4	2	2	5	7	2	5	7	5	2	2	1	7	1	1	1	1	1	1	2	2	1
**Overall guideline assessment**																											
1. Rate the overall quality of this guideline	6	6	4	5	6	6	3	5	2	4	3	3	3	5	2	4	2	3	4	4	2	4	4	1	2	2	2
2. I would recommend this guideline for use	YM	YM	YM	Y	Y	Y	N	YM	N	YM	YM	N	N	YM	N	YM	N	N	YM	YM	N	YM	YM	N	N	N	N

Table S8: Recommendations and decision for inclusion or exclusion

No.	CPG	Recommendation	Category	Decision	Summary recommendation number	Reason for exclusion
**Pain**						
1	AWMF	The management of analgesia, sedation and delirium should also be protocol-based in children according to the approach define goal - measure – treat*.	A1i, B2, E, B1	I	24	
2	AWMF	Age-appropriate, validated scoring systems should be used in children for therapy management and monitoring of analgesia, sedation and delirium.	A1i	I	2	
3	AWMF	If possible, children should self-report their pain themselves.	A1i	I	1	
4	AWMF	In children, behavioural characteristics such as facial expression, crying, motor activity, posture, activity, restlessness, apathy and appearance should be considered by the pain assessment as valid indicators of the presence of pain.	A1i	E		Isolated recommendation
5	AWMF	For children from the age of 4, the Faces Pain Scale - Revised is best used for self-assessment. From school age, the alternative use of numerical rating scales or visual analogue scales is also possible.	A1i	I	1	
6	AWMF	The Comfort-B Scale, the Children's and Infants Postoperative Pain Scale (CHIPPS, in German KUSS) or the FLACC scale should be used as validated and realizable assessment scale for the assessment of acute postoperative pain in non-ventilated infants and children up to 4 years of age.	A1i	I	2	
7	AWMF	The Comfort-B Scale is recommended for use for the assessment of acute postoperative pain in ventilated infants and children up to 4 years of age.	A1i	I	2	
8	AWMF	For the assessment of pain in children and adolescents who are unable to communicate due to severe cognitive impairment, the use of specially validated measurement instruments is recommended (e.g., Paediatric Pain Profile or Non-Communicating Children's Pain Checklist Revised, INRS).	A1i	I	30	
9	AWMF	In paediatric intensive care, patient-centered therapy strategies for analgesia, sedation, anxiety and delirium should be applied with individual targets of therapy goals*.	B2	I	19	
10	AWMF	Critically ill children in intensive care units should receive pain therapy adapted to the individual situation, regardless of the need for sedation.	B2	I?	19	
11	AWMF	Continuous intravenous infusion of an opioid should be used in neonatal and paediatric intensive care for severe pain.	B2	I	12	

12	AWMF	For severe pain, pain management in older children should be with an opioid in combination with a non-opioid.	B2	I	12	
13	AWMF	Local and regional peripheral and spinal analgesia should be considered in analgesic therapy.	B2	E		Isolated recommendation
14	AWMF	Patient-controlled analgesia (PCA) may be useful in children from about 5 years of age; parent- and/or caregiver-controlled analgesia may be used in children < 6 years of age.	B2	E		Isolated recommendation
15	AWMF	Additional pain reduction measures should be considered for acute, procedural pain of the newborn, such as the administration of oral sugar substances (e.g. glucose or sucrose), and the use of non-pharmacological measures (non-nutritive sucking, breastfeeding, facilitated tucking, kangaroo care, etc.).	B3	E		Isolated recommendation
16	ESPNIC	Use an age-appropriate tool to assess acute and prolonged pain i.e. the PIPP(-revised) in neonates and the COMFORT behaviour scale, FLACC or MAPS in critically ill children.	A1i	I	2	
17	ESPNIC	Pain assessment should take place routinely, depending on therapeutic goals, but at greater frequency (1–2 h) if the patient is receiving any analgesic infusion.	A1i	I	6	
18	ESPNIC	The effect (e.g. increasing or decreasing of a pump, bolus) of a drug should be re-evaluated depending on the drug's half-life.	A1i, B2	E		Isolated recommendation
19	ESPNIC	Validated assessment tools for pain, sedation, withdrawal syndrome and delirium should be integrated in pain and non-pain-related treatment protocols.	A1i, E, B1, B2, B3	I	24	
20	ESPNIC	Parent and family assessment of pain should be considered in pain assessment.	A1ii	E		Isolated recommendation
21	ESPNIC	Identify potential sources of pain and take appropriate actions.	B1, B2, B3	I	27	
22	ESPNIC	Pain assessment audits should take place regularly.	E	I	25	
23	NVP	It is recommended by the working group to agree on the use of pain assessment instruments based on the child's developmental level, ability to communicate and condition. From zero to four years, an observation scale is used (COMFORT neo, FLACC), supplemented by the VAS. From four years, the FLACC or self-reporting using the Faces Pain Scale-revisited (FPS-R) can be used and from seven years the VAS.	A1i, E	I	1,2	
24	NVP	It is recommended by the working group to agree on the use of pain assessment tools, their implementation and linking pain assessment to an existing treatment plan, using the VMS practice guide "Safe care in sick children" as a guide.	A1i, C, E, B2, B3	E	27	
25	NVP	Monitoring recommendation: In addition to pain score and respiratory checks, monitoring of respiration via respiratory/saturation monitor during intravenous morphine administration is necessary in patients:<br>- in the first month of life (at term): monitoring in intensive care, special care or recovery;	A1i	E		Isolated recommendation

		- aged one to six months, premature infants under the age of one year, with concomitant problems (respiratory, renal/liver and neuromuscular diseases) or with concomitant use of sedatives: monitoring on recovery and then on the ward;<br>- in children older than six months without the above-mentioned problems, who do not receive sedatives are administered, pain and sedation scoring and monitoring of respiration on the recovery and then on the ward is sufficient.				
26	NVP	The working group recommends hospital-level agreements on pain prevention in children.	B1, E	I	27	
27	NVP	Non-drug therapy is not recommended as prophylaxis for postoperative nausea and vomiting.	B1	E		Nausea and vomiting included in only one CPG
28	NVP	In patients at moderate to high risk of postoperative nausea and vomiting, it is advised to use two or three classes of antiemetics.	B1, B2	E		Nausea and vomiting included in only one CPG
29	NVP	Paracetamol is a safe and effective analgesic for mild pain that can be used from 28 weeks gestational age.	B2	I	17	
30	NVP	The doses of paracetamol in children should be adjusted for when poor general condition, hepatic and renal dysfunction, myopathy, when using medication with the same “pathway” and when used for longer than three days.	B2	I	17	
31	NVP	Diclofenac and ibuprofen are the most appropriate NSAID for children from the age of three/four months to control moderate pain, whether or not in combination with paracetamol and/or opioids. It reduces nausea and vomiting and has a low incidence of side effects.	B2	I	16	
32	NVP	Rectal administration of diclofenac seems to be preferred in the perioperative period.	B2	E		Isolated recommendation
33	NVP	Diclofenac and ibuprofen do not appear to increase the incidence of postoperative bleeding, requiring surgical management, do not increase the incidence of postoperative bleeding, but are contraindicated in children with coagulation disorders.	B2	E		Isolated recommendation
34	NVP	Diclofenac and ibuprofen can be used in children with asthma and do not affect bone healing (orthopedic procedures). Caution should be exercised in hepatic impairment and increased risk of renal failure and concomitant use of nephrotoxic agents.	B2	E		Isolated recommendation
35	NVP	Based on the limited literature available, the use of Diypyrone as an analgesic in children could be considered. More research is ongoing to determine the place of Dipyrone.	B2	E		Isolated recommendation
36	NVP	Tramadol can be used orally, rectally or intravenously in moderate pain in children from the age of one year, especially if NSAID are contraindicated.	B2	E		Isolated recommendation
37	NVP	Morphine is the opioid of choice in children for controlling severe pain after major surgery. Morphine administration via continuous infusion or by PCA is preferred, in combination with paracetamol and/or NSAID. Dosage and monitoring should take into account technique, age, weight and underlying suffering.	B2	I	12	

38	NVP	Oxycodone can be used as an alternative to morphine in children aged six years and over who do not need an intravenous access route, especially in the period following intravenous administration of opioids.	B2	E		Isolated recommendation
39	NVP	The study group believes that Esketamine should be carefully considered in children as an adjuvant in major surgery, if opioids lead to severe nausea and vomiting or if a chronic pain syndrome can be expected (major trauma or amputation).	B2	E		Isolated recommendation.
40	NVP	The working group believes that locoregional techniques can be used in children especially in those situations where the occurrence of respiratory depression due to the use of opioids poses additional risks (day care, prematures, neonates). More research is needed to demonstrate the benefits of regional analgesia over other forms of analgesia.	B2, B1	E		Isolated recommendation
41	NVP	The working group is of the opinion that locoregional techniques in children should be performed under general anesthesia, taking into account the basic safety rules.	B2	E		Isolated recommendation
42	NVP	Droperidol in low doses (0.625mg to 1.25 mg) can be used as a prophylactic in postoperative nausea and vomiting.	B2	E		Isolated recommendation.
43	NVP	In patients at moderate to high risk of postoperative nausea and vomiting, it is advised to use two or three classes of antiemetics.	B2, B1	E		Isolated recommendation.
44	NVP	For the treatment of postoperative nausea and vomiting, dexamethasone, droperidol and 5-HT3 antagonists are recommended. Metoclopramide is the least effective. Combination therapy is most effective.	B2	E		Nausea and vomiting included in only one CPG.
45	NVP	The working group believes that pediatric pain groups in the Netherlands should function in close collaboration with organized postoperative pain management, both in policy and implementation and in execution. In children, this multidisciplinary team includes: paediatric specialists (paediatrician/neonatologist, surgeons, paediatric anaesthesiologist) and paediatric nurses.	E, C	E		Isolated recommendation
46	SCCM	We suggest” that, in critically ill pediatric patients 6 years old and older who are capable of communicating, pain assessment via self-report be routinely performed using the Visual Analog Scale (VAS), Numeric Rating Scale, Oucher Scale, or Wong-Baker Faces pain scale.	A1i	I	1	
47	SCCM	We recommend” the use of either the Faces, Legs, Activity, Cry, and Consolability (FLACC) or COMFORT-Behavior (COMFORT-B) scales for assessing pain in non-communicative critically ill pediatric patients.	A1i	I	2	
48	SCCM	We recommend the use of observational pain assessment tools rather than vital signs alone for assessment of postoperative pain in critically ill pediatric patients.	A1i	I	2	
49	SCCM	We suggest the use of observational pain assessment tools rather than vital signs alone for assessment of procedure-related pain in critically ill pediatric patients.	A1i	I	2	
50	SCCM	We recommend that IV opioids be used as the primary analgesic for treating moderate to severe pain in critically ill pediatric patients.	B2	I	12	

51	SCCM	We recommend the addition of an adjunct nonsteroidal anti-inflammatory drug (NSAID) (IV or oral) to improve early postoperative analgesia in critically ill pediatric.	B2	I	16	
52	SCCM	We suggest the addition of an adjunct NSAID agent to decrease opioid requirements in the immediate postoperative period in critically ill pediatric patients (IV or oral).	B2	I	16	
53	SCCM	We suggest the addition of adjunct acetaminophen (IV or oral) to improve early postoperative analgesia in critically ill pediatric patients.	B2	I	17	
54	SCCM	We suggest the addition of adjunct acetaminophen (IV or oral) to decrease opioid requirements in the immediate postoperative period in critically ill pediatric patients.	B2, B1	I	17	
55	SCCM	We recommend that music therapy be offered to augment analgesia for critically ill postoperative pediatric patients.	B3	E		Isolated recommendation
56	SCCM	We suggest the use of protocolized sedation in all critically ill pediatric patients requiring sedation and/or analgesia during MV.	E, B2	I	24	
57	SARNEPI	We recommend regularly monitoring with validated tools the level of analgosedation of paediatric patients admitted to ICU*.	A1i	I	6	
58	SARNEPI	As a first-line strategy, we suggest optimizing analgesia using opiates and adopting alpha agonists as sedative agents, considering benzodiazepines a second-line*.	B2	I	12, 13	
59	SARNEPI	In difficult analgesia/sedation we suggest using ketamine, due to its good safety profile*.	B2	I	18	
60	SARNEPI	We suggest adopting protocols of analgesia and sedation to administer the minimal effective dose of analgesics and sedatives to reduce tolerance and the incidence of difficult analgesia/sedation. Furthermore, the daily interruption of sedation should be considered with caution*.	B2, E, B1, A1i	I	22	
61	SCCM	We suggest use of a standardized protocol for sedation/analgesia weaning to decrease duration of sedation taper and attenuate emergence of IWS.	B1, B2, E	I	21	
62	AWMF	Analgesia, sedation and delirium management in the ICU should conform to guidelines and be subject to quality assurance.	E	I	25	
63	AWMF	Provided that the intensive care nursing staff (specialist nursing standard) has special and qualified knowledge, experience and skills, the goal-oriented control of analgesia and sedation can be carried out by the nursing staff according to prescribed protocols and medical orders.	B2, E	I	24	
64	AWMF	With the aim of increasing treatment safety and facilitating decision- making, guideline-based internal hospital standards for analgesia, sedation and delirium therapy (including the use of sedation protocols) should be implemented.	E, C	I	25, 27	
65	NVP	It is recommended that an intravenous bolus be given before incision (to prevent early-stage hyperalgesia) combined with an infusion during and/or after the procedure.	B2	E		Isolated recommendation
**Sedation**						

1	AWMF	The Neonatal Pain, Agitation and Sedation Scale (N-PASS) or the COMFORTneo scale should be used to assess depth of sedation in preterm and term infants.	A1i	E		Isolated recommendation
2	AWMF	The Comfort-B Scale should be used for third-party assessment of sedation depth in infants and children.	A1i	I	3	
3	AWMF	Age-appropriate, validated scoring systems should be used in children for therapy management and monitoring of analgesia, sedation and delirium.	A1i, B2	I	1,2,3	
4	AWMF	The management of analgesia, sedation and delirium should also be protocol-based in children according to the approach define goal - measure – treat*.	A1i, B2, E	I	24	
5	ESPNIC	Use standardized sedation assessment tools with proven validity, reliability and clinical utility; the COMFORT behaviour scale.	A1i	I	3	
6	ESPNIC	The effect (e.g. increasing or decreasing of a pump, bolus) of a drug should be re-evaluated depending on the drug's half-life.	A1i, B2	E		Isolated recommendation
7	ESPNIC	Validated assessment tools for pain, sedation, withdrawal syndrome and delirium should be integrated in pain and non-pain-related treatment protocols.	A1i, E, B1, B2, B3	I	24	
8	ESPNIC	Together with the vital signs, the level of sedation must be assessed and documented every 4–8 h or as indicated by the sedation score or the child's clinical condition.	A1i	I	6	
9	SCCM	We recommend the use the Comfort-B Scale or the State Behavioral Scale (SBS) to assess level of sedation in mechanically ventilated pediatric patients.	A1i	I	3	
10	SCCM	We suggest the use of the Richmond Agitation-Sedation Scale (RASS) to assess level of sedation in mechanically ventilated pediatric patients.	A1i	I	3	
11	SCCM	We suggest that all pediatric patients requiring MV be assigned a target depth of sedation using a validated sedation assessment tool at least once daily.	A1i, E, B2	I	3,6	
12	SCCM	During the periextubation period when sedation is typically lightened, we suggest the following bundle strategies to decrease risk of inadvertent device removal: a) Assign a target depth of sedation at increasing frequency to adapt to changes inpatient clinical status and communicate strategies to reach titration goal, b) Consider a sedation weaning protocol, c) Consider unit standards for securement of endotracheal tubes and safety plan, d) Restrict nursing workload to facilitate frequent patient monitoring, decrease sedation requirements, and risk of self-harm.	A1i, B2, E, B1	I	21	
13	SARNePI	We recommend regularly monitoring with validated tools the level of analgosedation of paediatric patients admitted to ICU*.	A1i	I	6	
14	SARNePI	In children with developmental delay, we suggest adopting validated tools to monitor the level of sedation, the presence of delirium and withdrawal syndrome in ICU, considering their limitations and involving the caregivers.**	A1i, B1	I	30	
15	ESPNIC	Search for potential causes of non-pain-related distress/discomfort to take appropriate actions.	B1, B2, B3	E	27	

16	SCCM	We recommend minimizing benzodiazepine-based sedation when feasible in critically ill pediatric patients to decrease incidence and/or duration or severity of delirium.	B1, B2	I	20	
17	SCCM	We suggest strategies to minimize overall sedation exposure whenever feasible to reduce coma and the incidence and/or severity of delirium in critically ill children.	B1, B2	I	20	.
18	SCCM	We suggest use of a standardized protocol for sedation/analgesia weaning to decrease duration of sedation taper and attenuate emergence of IWS.	B1, B2, E	I	21	
19	AWMF	If permanent sedation is necessary, careful titration to the lowest possible dose *should be* carried out on the basis of regularly collected scores. A recommendation for a specific sedation concept in children cannot be made at present.	B2, B1	I	19	
20	AWMF	Midazolam can be used to sedate older, critically ill children who require intravenous sedation. This can be administered continuously.	B2	E		Midazolam included in only this CPG
21	AWMF	For sedation of paediatric ICU patients, continuous intravenous administration of alpha2-agonists (clonidine or dexmedetomidine) for decreasing nervous system activity (keeping vegetative) should be considered adjuvantly or as an alternative to sedation with midazolam.	B2, B1	E		Midazolam included in only this CPG
22	AWMF	In older, critically ill children, enteral sedatives should be used early if possible.	B2, B1	E		Isolated recommendation
23	AWMF	If sedation is necessary in individual cases in neonates, opioids should be used in preference to midazolam.	B2	E		Midazolam included in only this CPG
24	AWMF	In neonates and preterm infants, the use of chloral hydrate and phenobarbital should only be considered individually in justified individual cases and after careful risk/benefit assessment. especially with regard to cerebral development and a prolongation of the duration of ventilation.	B2	E		Isolated recommendation
25	AWMF	In paediatric intensive care, patient-centered therapy strategies for analgesia, sedation, anxiety and delirium should be applied with individual targets of therapy goals*.	B2, B3, B1, E	I	19	
26	SCCM	We suggest the use of alpha2-agonists as the primary sedative class in critically ill pediatric patients requiring MV.	B2	I	13	
27	SCCM	We recommend that dexmedetomidine be considered as a primary agent for sedation in critically ill pediatric postoperative cardiac surgical patients with expected early extubation.	B2	E		Dexmedetomidine is only including in this CPG
28	SCCM	We suggest the use of dexmedetomidine for sedation in critically ill pediatric postoperative cardiac surgical patients to decrease the risk of tachyarrhythmias.	B2, B1	E		Dexmedetomidine is only including in this CPG

29	SCCM	We suggest that continuous propofol sedation at doses less than 4 mg/kg/hr (67 μg/kg/min) and administered for less than 48 hours may be a safe sedation alternative to minimize the risk of propofol-related infusion syndrome (PRIS) development.	B2, B1	E		Propofol included only in this CPG
30	SCCM	Short term (< 48 hr) continuous propofol sedation may be a useful adjunct during the periextubation period to facilitate weaning of other analgosedative agents prior to extubation.	B2, B1	E		Propofol included only in this CPG
31	SCCM	We suggest consideration of adjunct sedation with ketamine in patients who are not otherwise at their predefined target sedation depth.	B2, A1i	I	18	
32	SCCM	The addition of daily sedation interruption (DSI) to sedation protocolization is not suggested due to lack of improvement in outcomes.	B2, E	I	22	
33	SARNePI	As a first-line strategy, we suggest optimizing analgesia using opiates and adopting alpha agonists as sedative agents, considering benzodiazepines a second-line*.	B2	I	12,13	
34	SARNePI	In difficult analgesia/sedation we suggest using ketamine, due to its good safety profile*.	B2	I	18	
35	SARNePI	We suggest adopting protocols of analgesia and sedation to administer the minimal effective dose of analgesics and sedatives to reduce tolerance and the incidence of difficult analgesia/sedation. Furthermore, the daily interruption of sedation should be considered with caution*.	B2, E, B1	I	22	
36	AWMF	Provided that the intensive care nursing staff (specialist nursing standard) has special and qualified knowledge, experience and skills, the goal-oriented control of analgesia and sedation can be carried out by the nursing staff according to prescribed protocols and medical orders.	B2, E	I	24	
37	SCCM	We suggest the use of protocolized sedation in all critically ill pediatric patients requiring sedation and/or analgesia during MV.	E, B2	I	24	
38	AWMF	Analgesia, sedation and delirium management in the ICU should conform to guidelines and be subject to quality assurance.	E	I	24, 25	
39	AWMF	With the aim of increasing treatment safety and facilitating decision-making, guideline-based internal hospital standards for analgesia, sedation and delirium therapy (including the use of sedation protocols) should be implemented.	E, C	I	25, 27	
40	AWMF	Permanent sedation of neonates should only be carried out in absolutely exceptional cases, e.g. in the case of life-threatening states of agitation that cannot be influenced in any other way, and with special consideration of the risk-benefit ratio.	B2	E		Only included in this CPG
**Delirium**						
1	SARNePI	We recommend regular monitoring delirium in critically ill children every day of the ICU stay, using validated tools.	A1i	I	7	
2	SARNePI	In children with developmental delay, we suggest adopting validated tools to monitor the level of sedation, the presence of delirium and withdrawal syndrome in ICU, considering their limitations and involving the caregivers.**	A1i	E		Isolated recommendation for delirium

3	SCCM	We recommend use of the preschool and pCAM-ICU (ps/pCAM-ICU) or the CAPD as the most valid and reliable delirium monitoring tools in critically ill pediatric patients.	A1i	I	4	
4	SCCM	We recommend routine screening for ICU delirium using a validated tool in critically ill pediatric patients upon admission through ICU discharge or transfer.	A1i	I	7	
5	ESPNIC	Use CAP-D as an instrument to assess paediatric delirium.	A1i	I	4	
6	ESPNIC	Together with the vital signs, delirium must be assessed and documented every 8–12 h (at least once per shift), 24–48 h after admission or as indicated by the delirium score of clinical condition of the child.	A1i	I	7	
7	ESPNIC	Validated assessment tools for pain, sedation, withdrawal syndrome and delirium should be integrated in pain and non-pain-related treatment protocols.	A1i, B1, B2, B3, E	I	24	
8	NVvP	Have further examination for PD by a competent doctor (e.g. child and adolescent psychiatrist) if there is evidence of change in the child's cognitive functions, perception, motor skills or behaviour, signalled by a multidisciplinary team (nurse, doctor, teaching assistant, etc.) and/or if parents indicate that they no longer recognise their child. If in doubt, consult with a competent doctor (e.g. child and adolescent psychiatrist).	A1i, A3	E		Isolated recommenation
9	NVvP	Diagnosis of PD in children aged 5 years and over who are not critically ill, neurologically impaired or intubated should be based on the criteria listed in the DSM-iv or using the pCAM-ICU.	A1i, A3	I	4	
10	NVvP	Diagnosis of PD in children aged three months to 5 years and/or are critically ill, neurologically impaired and/or intubated children, use the CAP-D, possibly supplemented by the parents' observation that they no longer recognise their child.	A1i, A3	I	4	
11	NVvP	In children with neurological disorders, include previous cognitive and neurological states in the assessment to determine whether the child also suffers from PD.	A1i, A3	E		Isolated recommendation
12	NVvP	A specialised paediatric nurse or expert physician (e.g. child and adolescent psychiatrist) screens daily for all the modifiable risk factors for PD listed in table 5.1 in a critically ill child and when phasing out sedatives or opioids (for use longer than five days).	A1i, B1, B2	I	7	
13	NVvP	Have nurses screen for PD three times a day in patients admitted to PICU for more than 48 hours using the PAED (revised, three-point scale), CAP-D or SOS-PD as a screening tool. This as part of routine measurement of (pain and) discomfort.	A1i, A3	I	4,7	
14	NVvP	Use a validated instrument in children on a PICU (e.g. COMFORT-B, and possibly RASS) to determine the degree of sedation/agitation/coma, before PD can be assessed.	A1i	I	2,3	
15	NVvP	Follow up the course of PD and the effect of treatment in children with a screening instrument, as there are no valid severity measurement instruments for children.	A1i, B4	E		Isolated recommendation
16	NVvP	When stopping sedatives, ask specifically about unpleasant dreams or thought processes in the child. If there are any, the child can be reassured. Talking to each other about these dreams and imaginings and making them tangible will make the child and his/her parents feel taken seriously and will result in anxiety reduction.	A1i, B3	E		Isolated recommendation

17	AWMF	The management of analgesia, sedation and delirium should also be protocol-based in children according to the approach define goal - measure – treat*.	A1i, B2, E	I	24	
18	AWMF	Regular targeted screening for delirious symptoms using a validated paediatric delirium score (e.g. pCAM-ICU, SOS-PD, CAP- D or PAED scale) should be performed.	A1i	I	4, 7	
19	AWMF	Age-appropriate, validated scoring systems should be used in children for therapy management and monitoring of analgesia, sedation and delirium.	A1i	I	2,3,4	
20	NVvP	For scientific research on PD, use the PAED, CAP-D, and the SOS-PD in children aged 0-16 years and the pCAM-ICU for research in children aged 5 years and over.	A1i	E		Don't meet the inclusion criteria
21	NVvP	Nurses and doctors should note these symptoms daily and systematically in the (nursing or medical) record; this concerns both neuropsychological (consciousness, orientation and memory disorders and hallucinations) and behavioural symptoms (agitation, motor restlessness, irritability) in (critically) ill children.	A2	I	7	
22	NVvP	The diagnosis of PD is made by a competent doctor (e.g. child and adolescent psychiatrist).	A3, A1i	E		Diagnosis included only in this CPG
23	NVvP	Have the diagnosis of PD confirmed and/or established by a competent doctor and, if in doubt, consult a competent doctor (e.g. child and adolescent psychiatrist).	A3, A1i	E		Diagnosis included only in this CPG
24	AWMF	Treatment of delirium in children should be symptom-oriented and include differential diagnosis for causal causes.	A3, B3, B2, A1i	E		Only included in this CPG
25	SARNePI	We recommend working on modifiable risk factors, particularly reducing the use of benzodiazepines.	B1, B2, E	I	20	
26	SCCM	Given low patient risk, and possible patient benefit to reduce the occurrence rate and/or decrease duration or severity of delirium, "we suggest" the following "nonpharmacologic strategies": optimization of sleep hygiene, use of interdisciplinary rounds, family engagement on rounds, and family involvement with direct patient care.	B1, B3	I	26, 28, 29	
27	SCCM	We suggest performing EM, when feasible, to reduce the development of delirium.	B1, B3	I	10	
28	SCCM	We recommend minimizing benzodiazepine-based sedation when feasible in critically ill pediatric patients to decrease incidence and/or duration or severity of delirium.	B1, B2,	I	20	
29	SCCM	We do not suggest routine use of haloperidol or atypical antipsychotics for the prevention of or decrease in duration of delirium in critically ill pediatric patients.	B1, B2,	I	11	
30	SCCM	We recommend a baseline electrocardiogram followed by routine electrolyte and QTc interval monitoring for patients receiving haloperidol or atypical antipsychotics	B1, B2	I	23	
31	ESPNIC	Search for potential sources of paediatric delirium and to take appropriate actions.	B1, B2, B3	E		Isolated recommendation.

32	NVvP	In children at risk of and with delirium, work together as a multidisciplinary team to align the most appropriate treatment for the child and his/her clinical picture.	B1, B2, B3, E	I	28	
33	NVvP	Have the child cared for by the same nurses as much as possible to provide as much uniformity in treatment and familiar faces as possible.	B1, E	I	10	
34	NVvP	Limit restraining the child as much as possible. Engage the parents or family to prevent the patient from injuring himself or removing accidentally necessary catheters and infusions.	B1, B3, E	I	10	
35	NVvP	Do not give additional drugs, such as antipsychotics, only for the prevention of PD.	B1, B2,	I	11	
36	SARNePI	We suggest basing the treatment of paediatric delirium on maximizing preventive bundles. Antipsychotic drugs may be used with careful consideration of contraindications.	B2, B3, E, B1	I	10	
37	SCCM	We suggest that in critically ill pediatric patients with refractory delirium, haloperidol or atypical antipsychotics be considered for the management of severe delirium manifestations, with consideration of possible adverse drug effects.	B2	I	11	
38	NVvP	Consider treatment of delirium with medication in children if non-medicated interventions do not have sufficiently rapid effect. This is particularly true when there is a lot of agitation or restlessness, with delusions or hallucinations, and also when delirium leads to danger for removal of IV lines or self-harm, in case of discomfort or stress in child and environment.	B2	I	14	
39	NVvP	Risperidone is the first choice when there are mild to moderate symptoms (moderate agitation) and there is a possibility for per-os- administration. This is especially true in cases of demonstrated sensitivity to extrapyramidal side effects.	B2	I	14	
40	NVvP	Haloperidol is the first choice in severe symptoms (agitation, psychotic symptoms) or if per-os administration is not possible.	B2	I	11	
41	NVvP	In case of non-response or adverse reactions to the first agent, switching agents should be considered.	B2,	E		Isolated recommendation.
42	NVvP	Weigh the risk of QT prolongation when starting antipsychotics and use in the presence of risk factors and in high-risk groups monitoring by ECG.	B2, B1	I	23	
43	AWMF	In paediatric intensive care, patient-centered therapy strategies for analgesia, sedation, anxiety and delirium should be applied with individual targets of therapy goals*.	B2, B3	E		Isolated recommendation
44	AWMF	Alpha2 agonists should be used as medication.	B2	E		Isolated recommendation.
45	NVvP	Have parents or a family member continuously present with a child with delirium or a high risk of its occurrence. Explain to the parents the importance of this.	B3, D2, B1, A1i, A1ii	I	29	
46	NVvP	Involve parents as much as possible in the care of the child with PD.	B3, E	I	29	
47	NVvP	Consider safety-oriented interventions (bed rails, anti-slip measures, physical supervision).	B3	I	10	

48	NVvP	Apply fixation, preferably, not when there are alternatives (especially physical supervision).	B3, E	I	10	
49	NVvP	Consider the following interventions: – Offer parental presence through rooming-in or recording parents' voices; – promote the child's orientation (staff name and function, photos, music and toys from home, calendar, whiteboard, glasses, hearing aid, dimmed light in the room at night); – Have the child cared for by the same nurses as much as possible to give as much uniformity of approach/treatment as possible and ensure familiar faces. Take into account impaired attention and memory functions (simple sentences, repeating information); – avoid overstimulation from noise, draughts, light, too many people. Earplugs can be helpful here. Do not hold conversations at the bedside. If necessary, move the child to a quieter (hyperactive delirium) or more stimulating (hypoactive delirium) environment; – Mobilise the child (physiotherapy and nurses); – take developmental level into account when communicating; – Improve day-night rhythm by offering activities, changing daylight, among other things; – Support dyspraxia, dysphasia and other factors that make communication difficult with aids (writing pad, pointer card, electronic resources).	B3, E, B1	I	10	
50	AWMF	Therapy should be non-pharmacological and with psychosocial interventions.	B3, E	I	10	
51	NVvP	Hold an evaluation interview on the course of delirium before the patient's discharge. This should discuss the course of the process and consider the perception and risk of recurrence in case of a new triggering factor. Make arrangements for aftercare.	B4, B5, E	E		Isolated recommendation.
52	NVvP	Given the study by Gillian Colville and others, it is recommended that children and adolescents who have gone through a PD, together with their parents, continue to be monitored by a child and adolescent psychiatrist or child psychologist at low frequency for at least six months. This given the risk of PTSD, as well as the risk of cognitive problems and based on evidence from the scarce literature in children, but the rich literature in adults and the elderly.	B4	E		Isolated recommendation.
53	NVvP	Provide continuous, proper continuing education for doctors and nurses on all facets of delirium to properly inform the parents and child and provide them with proper guidance.	Di, E, B1, B2, B3, D2	E		Isolated recommendation.
54	NVvP	Provide in-service training for treating physicians and nurses responsible for treating delirium on the expected side effects of antipsychotics, especially extrapyramidal side effects. Herein lies a role for the (paediatric) psychiatrist or doctor who regularly prescribes this medication.	Di, E	E		Isolated recommendation.
55	NVvP	Prepare the child and his parents well for interventions on the child so that the child is less anxious. Good preparation leads to anxiety reduction in the child and parents and thus reduces the risk of delirium.	D2, B1	E		Isolated recommendation.

56	NVvP	Provide both oral and written information about the possible occurrence of delirium to the patient and his parents in the case of a child at increased risk of delirium. See also Appendix 2 Leaflet.	D2, B1	E		Isolated recommendation.
57	NVvP	Offer family education about delirium (orally and in writing through an information leaflet).	D2, B3	I	29	
58	AWMF	Analgesia, sedation and delirium management in the ICU should conform to guidelines and be subject to quality assurance.	E	I	25	
59	AWMF	With the aim of increasing treatment safety and facilitating decision-making, guideline-based internal hospital standards for analgesia, sedation and delirium therapy (including the use of sedation protocols) should be implemented.	E, C	I	24,25	
60	NVvP	Have nurses on a (medium care) paediatric ward use a screening tool in high-risk children (e.g. POST-IC) for screening for PD for 72 hours.	A1i	E		Isolated recommendation
61	SCCM	We suggest strategies to minimize overall sedation exposure whenever feasible to reduce coma and the incidence and/or severity of delirium in critically ill children.	B1, B2	I	20	
**Iatrogenic withdrawal**						
1	SARNePI	We recommend regular monitoring withdrawal symptoms in critically ill children treated with analgesics and/or sedatives longer than 72 h, adopting validated tools.	A1i	I	8	
2	SARNePI	In children with developmental delay, we suggest adopting validated tools to monitor the level of sedation, the presence of delirium and withdrawal syndrome in ICU, considering their limitations and involving the caregivers**.	A1i	E		Could not be combined for IWS, only one recommendation
3	SCCM	We recommend use of either the Withdrawal Assessment Tool-1 (WAT-1) or Sophia Observation Scale (SOS) for the assessment of IWS due to opioid or benzodiazepine withdrawal in critically ill pediatric patients.	A1i	I	5	
4	SCCM	We suggest routine IWS screening after a shorter duration (3–5 d) when higher opioid or benzodiazepine doses are used.	A1i	I	8	
5	ESPNIC	Use standardized IWS assessment instruments with proven clinical utility, validity and reliability in infants and children; WAT-1 or the SOS.	A1i	I	5	
6	ESPNIC	Reassess for symptoms of withdrawal after treatment interventions.	A1i	E		Isolated recommendation.
7	ESPNIC	Validated assessment tools for pain, sedation, withdrawal syndrome and delirium should be integrated in pain and non-pain-related treatment protocols.	A1i	I		
8	AWMF	Opioid withdrawal syndrome should be considered after continuous therapy. Measurement instruments for assessing opioid withdrawal can be the Finnegan score in neonates or the SOS-PD (2018) or the WAT-1 in older children and adolescents.	A1i	I	5, 8	
9	AWMF	The possibility of a sedative withdrawal syndrome (especially seizures) should be considered after continuous therapy. As Measurement tool for assessing sedative	A1i	I	5, 8	

		withdrawal, the Finnegan score that can be used for newborns and for older children and adolescents, the SOS-PD or the WAT-1.				
10	SCCM	Until a validated screening tool is developed, monitoring for IWS from alpha2-agonists should be performed using a combination of associated symptoms (unexplained hypertension or tachycardia) with adjunct use of a validated benzodiazepine or opioid screening tool.	A1i	E		Isolated recommendation.
11	ESPNIC	The potential risk of opioid and/or benzodiazepine iatrogenic withdrawal syndrome should be considered after 5 days of continuous administration of these drugs.	B1	I	8	
12	SARNePI	We recommend to working on modifiable risk factors of WS, particularly avoiding weaning higher than a daily reduction of 20% respect on the initial dose.	B1	I	21	
13	SCCM	We suggest use of a standardized protocol for sedation/analgesia weaning to decrease duration of sedation taper and attenuate emergence of IWS.	B1	I	21	
14	SARNePI	We recommend treating withdrawal symptoms with additional boluses of the drug considered to be responsible for the symptoms and modifying the weaning plan.	B2	I	15, 21	
15	SCCM	We suggest that opioid related IWS be treated with opioid replacement therapy to attenuate symptoms, irrespective of preceding dose and /or duration or opioid exposure.	B2	I	15	
16	SCCM	Benzodiazepine-related IWS should be treated with benzodiazepine replacement therapy to attenuate symptoms, irrespective of preceding dose and/or duration of benzodiazepine exposure.	B2	I	15	
17	SCCM	Alpha2-agonist–related IWS should be treated with IV and/or or enteral alpha2-agonist replacement therapy to attenuate symptoms, irrespective of preceding dose and/or duration of alpha2-agonist exposure.	B2	I	15	
18	AWMF	To avoid an opioid withdrawal syndrome, opioids should be phased out after continuous therapy if possible and supplemented with alpha2 agonists.	B2	I	21	
19	AWMF	To avoid a sedative withdrawal syndrome, these drugs should be specifically phased out after continuous therapy, taking risk factors into account (e.g. long sedative doses), and alpha2 agonists should be used additively.	B2	I	21	
20	AWMF	Analgesia, sedation and delirium management in the ICU should conform to guidelines and be subject to quality assurance.	E	I	25	
**Other**						
1	SARNePI	We recommend adopting in all paediatric patients admitted to ICU strategies to prevent sleep alterations, particularly non-pharmacologic ones (relaxing techniques, parental involvement, control of environmental factors).	B3, E,	I	26, 29	
2	SARNePI	We recommend explaining to parents the meaning of analgesia and sedation and off-label drugs. If analgesia and sedation lasted more than 48 h, we recommend informing parents about the risk of withdrawal syndrome and delirium development.	D2, E	I	29	

3	SCCM	We suggest facilitation of parental or caregiver presence in the PICU during routine care and interventional procedures to a) provide comfort to the child, b) decrease parental levels of stress and anxiety and c) increase level of satisfaction of care.	B3, E	I	29	
4	SCCM	We suggest offering patients the use of noise reducing devices/ or behavioral changes to reduce excessive noise and therefore improve sleep hygiene and comfort, in critically ill pediatric patients.	B1, E, B3	I	26	
5	SCCM	We suggest offering patients the use of noise reducing devices such as ear plugs or headphones to reduce the impact of nonmodifiable ambient noise.	B1, E, B3	I	26	
6	SCCM	We suggest performing EM to minimize the effects of immobility in critically ill pediatric patients.	B3, B1	E		Only included in 1 CPGs
7	SCCM	We suggest the use of a standardized EM protocol that outlines readiness criteria, contraindications, developmentally appropriate mobility activities and goals, and safety thresholds guided by the multidisciplinary team and family decision-making.	E, B1	E		Only included in 1 CPGs
8	AWMF	In children, attention should be paid to any correctable environmental factor (e.g. noise suppression by music) and caring treatment should be ensured.	B1, B3	E		Only included in 1 CPGs
9	AWMF	A normal sleep pattern should be encouraged, especially adequate lighting, reduction of noise and an adapted day-night rhythm for the patient should be taken into account.	B1, E, B3	I	26	
10	AWMF	Analgesia, sedation and delirium management in the ICU should conform to guidelines and be subject to quality assurance.	E	I	25	
11	AWMF	Provided that the intensive care nursing staff (specialist nursing standard) has special and qualified knowledge, experience and skills, the goal-oriented control of analgesia and sedation can be carried out by the nursing staff according to prescribed protocols and medical orders.	B2, E	I	24, 25	
12	AWMF	With the aim of increasing treatment safety and facilitating decision-making, guideline-based internal hospital standards for analgesia, sedation and delirium therapy (including the use of sedation protocols) should be implemented.	E, C	I	24, 25	
13	AWMF	For consistent implementation of guidelines or standards, regular training of staff in their application should take place.	Di, C	I	23	

I : Inclusion; E : Exclusion; ☐ = Duplicated in other sections ▒ = not applicable

Categories:

A. Assessment: A1i) Assessment/screening by HCPs, A1ii) Assessment by families, A2) Documentation, A3) Diagnosis
B. Management/intervention: B1) Prevention, B2) Pharmacological, B3) Non-pharmacological, B4) Follow-up/discharge, B5) Planning, B6) Diagnosis
C. Implementation
D. Education
E. Organization

Summary recommendations:

1. Children, as young as 4 years of age, but certainly from the age of 6, should be asked to self-report their pain using the VAS or NRS whenever possible.
2. Use age-appropriate scales to assess pain in non-communicative critically ill children, either the FLACC, or MAPS scales- and the COMFORT-B for mechanically ventilated.
3. Use age-appropriate scales to assess sedation in non-communicative critically ill children (COMFORT-B, RASS).

4. Use age-appropriate scales for monitoring delirium in critically ill children (CAPD or pCAM-ICU, SOS-PD, PAED).
5. Use age-appropriate scales to assess IWS in critically ill children (SOS/SOS-PD, WAT-1).
6. Ensure regular monitoring of analgosedation levels in pediatric patients in the PICU. Increase pain assessment to every 1–2 hours for patients receiving analgesic infusion. Assess and document sedation every 4–8 h or as needed based on sedation scores or the child's clinical condition.
7. Regular and routine screening for delirium in pediatric patients in the PICU. Assess and document every 8–12 h (at least once per shift), 24–48 h after admission or as indicated by the child's clinical condition.
8. Regular and routine screening for IWS in pediatric patients in the PICU after 3 – 5 days of continuous therapy with opioids or benzodiazepines.
9. In pediatric patients at risk or with delirium, promote parental involvement by providing clear explanations of its importance. Encourage active participation during rounds, involve them in direct patient care, and ensure their continuous presence.
10. Implement delirium bundles to prevent delirium in pediatric patients, which should include promoting parental presence or voice recording, orientating the child with familiar items and functional aids, ensuring consistent nursing care with staff introductions, adapting communication using simple sentences and aids, minimizing overstimulation from noise, light, and excessive people, and establishing a structured day-night schedule. Additionally, prioritize early mobilization and reserve the use of restraints as a last resort, after all other safety-oriented alternatives such as bed rails, anti-slip measures, and physical supervision have failed.
11. The routine use of haloperidol or atypical antipsychotics for the prevention of pediatric delirium is not recommended.
12. As a first-line approach, IV opioids, specifically morphine, should be used for the treatment of moderate to severe pain in critically ill pediatric patients. Morphine is the preferred opioid for managing severe pain after surgery.
13. Consider adopting alpha2-agonists as the primary sedative class in critically ill pediatric patients requiring mechanical ventilation as the first-line approach.
14. In critically ill pediatric patients with refractory delirium, medication should be considered if non-pharmacological interventions fail to provide rapid relief, especially from agitation, restlessness, delusions, hallucinations, or risks to the patient's safety. Risperidone is the preferred choice for mild to moderate symptoms if oral administration is possible and there is sensitivity to extrapyramidal side effects. Haloperidol is the preferred choice for severe symptoms or when oral administration is not possible.
15. IWS should be treated with an additional bolus of the same medication which caused the symptoms.
16. Adjunct NSAIDs (IV or oral) can be added to improve early postoperative analgesia in critically ill patients. Particularly ibuprofen or diclofenac for children aged three and older.
17. Adjunct acetaminophen (IV or oral) can improve mild postoperative pain in critically ill pediatric patients. It can be used from 28 weeks gestational age.
18. In difficult to sedate, consider using ketamine due to its good safety profile.
19. Patient-centered pain and sedation management should be applied in the PICU with targeted therapy for each patient to decrease opioid requirements and for ensuring that the minimal effective dose is administered. Medications should be adjusted for poor general condition, hepatic and renal dysfunction, myopathy, when using medication with the same "pathway" and when used for longer than three days.
20. Minimize benzodiazepine use to reduce the incidence, duration, and severity of delirium in critically ill pediatric patients.
21. Utilize a standardized sedation/analgesia weaning protocol, gradually reducing the dose by a maximum of 20% per day from the initial dose. Modify the weaning plan as needed and consider supplementing with alpha2 agonists when appropriate.
22. Daily sedation interruption is not suggested and should be used with caution.
23. Weighing the risk of QT prolongation when starting antipsychotics, especially in high-risk groups and those with risk factors, and implementing monitoring through baseline electrocardiogram and routine electrolyte and QTc interval monitoring for patients receiving haloperidol or atypical antipsychotics.
24. Protocolization of pain, sedation, delirium, and weaning (to prevent IWS), incorporating measurement instruments, to manage these conditions, including medications to reduce adverse outcomes.
25. Incorporate pain, sedation, and delirium management into unit guidelines and procedures, and conduct regular quality audits of adherence and effectiveness, along with staff training,
26. Optimize the PICU environment by providing earplugs and headphones to reduce noise and promote better sleep.
27. Identify and address potential sources of pain in children by establishing hospital-level agreements on pain prevention.
28. In children at risk of and with delirium, collaborate as a multidisciplinary team to align the most appropriate treatment for the child and their clinical picture, utilizing interdisciplinary rounds.
29. Parental involvement in the PICU should include: promoting understanding of analgesia, sedation, and off-label drug use, informing about the risks of withdrawal syndrome and delirium, and facilitating presence during routine care and interventional procedures to enhance child comfort, reduce parental stress and anxiety, and improve satisfaction with care.

30.	For children with developmental delays, the use specially validated measurement instruments, such as the Paediatric Pain Profile or Non-Communicating Children's Pain Checklist Revised, INRS, for pain assessment, while considering their limitations and involving the caregivers

Table S9: Synthesized pain recommendations – consistency across CPGs for SoR,and CoE, and review of evidence relevance and support

Overall Recommendations			Supporting Information							
Synthesis			Recommendations			Evidence				Other CPGs with similar recommend-ations
Summary recommendation	GoR	LoE	Recommendations	SoR	C or D GoR	CoE	References	Relevance to recommendation	C or D COE	
**1. ASSESSMENT**										
**a. Self-report**										
**It is recommended that children should be asked to self-report their pain whenever possible. As young as 4 years of age can use the FPS-R, and from 6 years of age can use the VAS or NRS.**	**Weak**	**Low**	We suggest that, in critically ill pediatric patients 6 years old and older who are capable of communicating, pain assessment via self-report be routinely performed using the Visual Analog Scale (VAS), Numeric Rating Scale, Oucher Scale, or Wong-Baker Faces pain scale. **(SCCM)**	Conditional	D	Low	Beltramini et al., 2017<br>Huguet et al., 2010<br>Stinson et al., 2006	Y-F<br>Y-F<br>Y-F	D	ASPNM<br>RNAO§<br>SFAR<br>APM & ASA§<br>SARNePI-M<br>ANZCA<br>APA<br>CMA<br>ÖGARI<br>PSAIT§
			It is recommended by the working group that from four years, self-reporting using the Faces Pain Scale-revisited (FPS-R) can be used and from seven years the VAS. **(Dutch-P)***	NI		A1,C,D	**FPS-R:**<br>Tovar et al., 2010<br>**Self-report:**<br>Van Dijk, 2001<br>Breau et al., 2009<br>VWS, 2011	Y-F<br>UC<br>Y-F<br>UC		
			If possible, children should self-report their pain themselves	A		2b, 3b, 5	Harris et al., 2016<br>Rajasagaram et al., 2009<br>Shavit et al., 2008	Y-P<br>Y-F<br>Y-F		
			For children from the age of 4, the FPS-R is best used for self-assessment. From school age, the alternative use of NRS or VAS is also possible **(AWMF)**			3a, 3b	**FPS-R:**<br>Hicks et al., 2001<br>**NRS or VAS:**<br>Birnie et al., 2019	Y-F<br>Y-F		
**b. Observational scales**										
**We recommend the use of age-appropriate scales to assess pain in non-communicative critically ill**	**Strong**	**Moderate**	We recommend" the use of either FLACC or COMFORT-B scales for assessing pain in non-	Strong	C	Moderate	LaFond et al., 2016<br>Beltramini et al., 2017	N-n<br>Y-F<br>N-n (self-report scales)	C	SARNePI –M<br>SFAR<br>APS/ASRAPM & ASA

**children, either the FLACC or the COMFORT-B scales**			communicative critically ill pediatric patients. **(SCCM)**				Huguet et al., 2010<br>Dorfman et al., 2014<br>Ahlers et al., 2008<br>**FLACC :**<br>Manworren et al., 2003<br>Voepel-Lewis et al., 2010<br>Malviya et al., 2006<br>**CB :**<br>Ambuel et al., 1992<br>van Dijk et al., 2000<br>Ista et al., 2005	Y-F<br>N-n (adult BPS/VAS/NRS<br>Y-F<br>Y-F<br>Y-P<br>Y-P<br>Y-P<br>Y-F		SSPM
			Use an age-appropriate tool to assess acute and prolonged pain i.e. COMFORT-B scale, FLACC or MAPS in critically ill children **(ESPNIC)***	A		NI	van Dijk et al., 2012<br>Johansson et al., 2009<br>de Jong et al., 2010<br>Dorfman et al., 2014<br>**FLACC** :<br>Merkel et al., 1997<br>Voepel-Lewis et al., 2010<br>**CB :**<br>Ambule et al., 1992<br>van Dijk et a., 2000<br>van Dijk et al., 2002<br>van Dijk et al., 2001<br>Bai et al., 2012<br>Boerlage et al., 2012<br>Valkenburg et al., 2011	Y-P<br>Y-F<br>Y-P (burn)<br>Y-F<br>Y-F<br>Y-F<br>Y-P<br>Y-P<br>?-P<br>?-n<br>Y-P (cardiac surgery)<br>?-n<br>?-P (Downs Syndrom)<br>Y-F<br>Y-F		

							**MAPS :**<br>Ramelet et al., 2007<br>Ramelet et al., 2007			
			It is recommended to use pain assessment instruments based on the child's developmental level, ability to communicate and condition. From zero to four years, an observation scale is used (FLACC) **(Dutch-P)***	NI		Level 4	**FLACC:**<br>Ramelet et al., 2007<br>Bringuier et al., 2009<br>Baeyer et al., 2007<br>Malviya et al., 2006	N-n<br>Y-P<br>Y-F<br>Y-Y		
			We recommend regularly monitoring with validated tools the level of analgosedation of paediatric patients admitted to ICU **(SARNePI)**	Strong		NI	Harris et al., 2016<br>Zuppa & Curley 2017<br>**CB :**<br>Ista et al., 2005<br>Boerlage et al., 2015<br>**RASS :**<br>Kerson et al., 2016<br>**FLACC :**<br>Voepel-Lewis et al., 2002	Y-P<br>Y-P<br>Y-F<br>?-P<br>Y-F<br>Y-F		
			Age-appropriate, validated scoring systems should be used in children for therapy management and monitoring of analgesia **(AWMF)**	A		A1, A2	Harris et al., 2016<br>Chou et al., 2016<br>Lim & Godambe, 2017<br>Gai et al., 2020<br>Baarslag et al., 2017	Y-P<br>Y-P<br>Y-P<br>Y-F<br>Y-F		
			The Comfort-B Scale, the Children's and Infants Postoperative Pain Scale	A		1a, 2b, 3b	Harris et al., 2016<br>**CB:**	Y-F<br>Y-F		

			(CHIPPS, in German KUSS) or the FLACC scale should be used as validated and realizable assessment scale for the assessment of acute postoperative pain in non-ventilated infants and children up to 4 years of age. **(AWMF)**				Boerlage et al., 2015<br>van Dijk et al., 2000<br>**FLACC:**<br>Voepel-Lewis et al., 2002<br>**KUSS:**<br>Buttner et al., 1990	Y-P<br>Y-F<br>Y-F		
			The Comfort-B Scale is recommended for use for the assessment of acute postoperative pain in ventilated infants and children up to 4 years of age. **(AWMF)**	A		1a, 1b, 2b, 3b	Harris et al., 2016<br>Boerlage et al., 2015<br>van Dijk et al., 2000	Y-F<br>Y-F<br>Y-P (Comfort scale)		
**c. Routine assessment**										
**Regularly monitoring the level of analgosedation of pediatric patients in the PICU, with pain assessment taking place routinely depending on therapeutic goals but at a great frequency (1-2 hours) if a patient is receiving any analgesic infusion**	**Conditional**	**Low**	We recommend regularly monitoring with validated tools the level of analgosedation of paediatric patients admitted to ICU* **(SARNePI)**	Strong	D	NI	Harris et al., 2016<br>Zuppa & Curley 2017	Y-P<br>N-n	NA	SSMP
			Pain assessment should take place routinely, depending on therapeutic goals, but at greater frequency (1–2 h) if the patient is receiving any analgesic infusion **(ESPNIC)**	D		NI	None			
**2. ORGANIZATIONAL**										
**Protocolized care**										
**Protocolization of pain, sedation, delirium should be adopted to manage these conditions, including medications to reduce adverse outcomes**	**Weak**	**Low**	The management of analgesia, sedation and delirium should also be protocol-based in children according to the defined goal - measurement - treatment approach. **(AWMF)**	A		5	Michel et al., 2017<br>Gaillard-Le Roux et al., 2017<br>Fenn & Plake, 2017<br>Garcia Guerra et al., 2016<br>Motta et al., 2016<br>Tabacco et al., 2017<br>Cunningham & Vogel, 2019	?-P.<br>?-P (sedation score)<br>.<br>N-n (survey on practice)<br>N-n (SR sedation protocols)<br>N-n (survey on practices)<br>.<br>.<br>?-P		

							Solodiuk et al., 2019<br>Neunhoeffer et al., 2017<br>Neunhoeffer et al., 2015<br>Dreyfus et al., 2017<br>Shah & Siu, 2019<br>Sanavia et al., 2019<br>Curley et al., 2015<br>Amirnovin et al., 2018	?-P<br>?-P (sedation score)<br>.<br>.<br>Y-P<br>N-n (r/t withdrawal)		
			We suggest adopting protocols of analgesia and sedation to administer the minimal effective dose of analgesics and sedatives to reduce tolerance and the incidence of difficult analgesia/sedation. **(SARNePI)***	Moderate		NI	Lucas et al., 2016<br>Curley et al., 2015<br>Deeter et al., 2011<br>Neunhoeffer et al., 2015<br>Neunhoeffer et al., 2017<br>Sanavia et al., 2019<br>Dreyfus et al., 2017<br>Gaillard-Le Roux et al., 2017<br>Yaghami et al., 2016	.<br>Y-F<br>Y-F<br>Y-F<br>Y-F<br><br>Y-F<br>Y-F<br><br>Y-P		

SoR = summary of recommendations; CoE = certainty of evidence; C or D = convergent or divergent; NI = not indicated; UC = unable to classify (reference not available for review).
§ - part of the body of evidence, inferred in the recommendation but not explicitly stated
*Recommendations are adapted to include only information relevant to each summarized recommendation and typical PICU population. As an example, the original ESNICP recommendation for observational scales includes the PIPP for neonates, this was removed.
Text in green = evidence that is similar across >2 recommendations
RELEVANCE TO RECOMMENTATION: Level of relatedness: Y = yes, N = no, ? = mixed, **Level of support**: P = partially, F = fully, n = none, R = refutes/contradicts
